# Placebo effects in randomized trials of pharmacological and neurostimulation interventions for mental disorders: An umbrella review

**DOI:** 10.1038/s41380-024-02638-x

**Published:** 2024-06-24

**Authors:** Nathan T. M. Huneke, Jay Amin, David S. Baldwin, Alessio Bellato, Valerie Brandt, Samuel R. Chamberlain, Christoph U. Correll, Luis Eudave, Matthew Garner, Corentin J. Gosling, Catherine M. Hill, Ruihua Hou, Oliver D. Howes, Konstantinos Ioannidis, Ole Köhler-Forsberg, Lucia Marzulli, Claire Reed, Julia M. A. Sinclair, Satneet Singh, Marco Solmi, Samuele Cortese

**Affiliations:** 1 Clinical and Experimental Sciences, Faculty of Medicine, University of Southampton, Southampton, UK; 2 Southern Health NHS Foundation Trust, Southampton, UK; 3 University Department of Psychiatry and Mental Health, University of Cape Town, Cape Town, South Africa; 4 School of Psychology, University of Nottingham Malaysia, Semenyih, Malaysia; 5 Centre for Innovation in Mental Health, School of Psychology, Faculty of Environmental and Life Sciences, University of Southampton, Southampton, UK; 6 Clinic of Psychiatry, Social Psychiatry and Psychotherapy, Hannover Medical School, Hanover, Germany; 7 Department of Child and Adolescent Psychiatry, Charité Universitätsmedizin Berlin, Berlin, Germany; 8 Department of Psychiatry, Zucker Hillside Hospital, Northwell Health, Glen Oaks, NY USA; 9 Department of Psychiatry and Molecular Medicine, Zucker School of Medicine at Hofstra/Northwell, Hempstead, NY USA; 10 Center for Psychiatric Neuroscience, Feinstein Institute for Medical Research, Manhasset, NY USA; 11 Faculty of Education and Psychology, University of Navarra, Pamplona, Spain; 12 School of Psychology, Faculty of Environmental and Life Sciences, University of Southampton, Southampton, UK; 13 Université Paris Nanterre, DysCo Lab, F-92000 Nanterre, France; 14 Université de Paris, Laboratoire de Psychopathologie et Processus de Santé, F-92100 Boulogne-Billancourt, France; 15 Department of Sleep Medicine, Southampton Children’s Hospital, Southampton, UK; 16 Department of Psychosis Studies, Institute of Psychiatry, Psychology & Neuroscience, King’s College London, London, UK; 17 H Lundbeck A/s, Iveco House, Watford, UK; 18 Institute of Clinical Sciences (ICS), Faculty of Medicine, Imperial College London, London, UK; 19 Department of Clinical Medicine, Aarhus University, Aarhus, Denmark; 20 Psychosis Research Unit, Aarhus University Hospital–Psychiatry, Aarhus, Denmark; 21 Department of Translational Biomedicine and Neuroscience (DIBRAIN), University of Studies of Bari “Aldo Moro”, Bari, Italy; 22 Department of Psychiatry, University of Ottawa, Ottawa, ON Canada; 23 Department of Mental Health, Ottawa Hospital, Ottawa, ON Canada; 24 Ottawa Hospital Research Institute (OHRI) Clinical Epidemiology Program, University of Ottawa, Ottawa, ON Canada; 25 School of Epidemiology and Public Health, Faculty of Medicine, University of Ottawa, Ottawa, ON Canada; 26 Solent NHS Trust, Southampton, UK; 27 DiMePRe-J-Department of Precision and Regenerative Medicine-Jonic Area, University “Aldo Moro”, Bari, Italy; 28 Hassenfeld Children’s Hospital at NYU Langone, New York University Child Study Center, New York, NY USA

**Keywords:** Psychiatric disorders, Drug discovery, Neuroscience

## Abstract

There is a growing literature exploring the placebo response within specific mental disorders, but no overarching quantitative synthesis of this research has analyzed evidence across mental disorders. We carried out an umbrella review of meta-analyses of randomized controlled trials (RCTs) of biological treatments (pharmacotherapy or neurostimulation) for mental disorders. We explored whether placebo effect size differs across distinct disorders, and the correlates of increased placebo effects. Based on a pre-registered protocol, we searched Medline, PsycInfo, EMBASE, and Web of Knowledge up to 23.10.2022 for systematic reviews and/or meta-analyses reporting placebo effect sizes in psychopharmacological or neurostimulation RCTs. Twenty meta-analyses, summarising 1,691 RCTs involving 261,730 patients, were included. Placebo effect size varied, and was large in alcohol use disorder (*g* = 0.90, 95% CI [0.70, 1.09]), depression (*g* = 1.10, 95% CI [1.06, 1.15]), restless legs syndrome (*g* = 1.41, 95% CI [1.25, 1.56]), and generalized anxiety disorder (*d* = 1.85, 95% CI [1.61, 2.09]). Placebo effect size was small-to-medium in obsessive-compulsive disorder (*d* = 0.32, 95% CI [0.22, 0.41]), primary insomnia (*g* = 0.35, 95% CI [0.28, 0.42]), and schizophrenia spectrum disorders (standardized mean change = 0.33, 95% CI [0.22, 0.44]). Correlates of larger placebo response in multiple mental disorders included later publication year (opposite finding for ADHD), younger age, more trial sites, larger sample size, increased baseline severity, and larger active treatment effect size. Most (18 of 20) meta-analyses were judged ‘low’ quality as per AMSTAR-2. Placebo effect sizes varied substantially across mental disorders. Future research should explore the sources of this variation. We identified important gaps in the literature, with no eligible systematic reviews/meta-analyses of placebo response in stress-related disorders, eating disorders, behavioural addictions, or bipolar mania.

## Introduction

A placebo is an ‘inactive’ substance or ‘sham’ technique that is used as a control for assessing the efficacy of an active treatment [1]. However, study participants in a placebo control group may experience considerable symptom improvements - a ‘placebo response’ [1–3]. Statistical artifacts or non-specific effects account for some of the placebo response. For example, many individuals seek treatment and are enrolled in clinical trials while their symptoms are at their worst. Their symptoms will gradually return to their usual severity (‘regression to the mean’), giving the appearance of a placebo response [4]. Further, it has been suggested that the placebo response is exacerbated due to unreliable ratings as well as baseline symptom severity inflation if raters are aware of severity criteria for entry to a trial [5, 6]. Other potential sources of apparent placebo responses include sampling biases caused by the withdrawal of the least improved patients in the placebo arm, non-specific beneficial effects resulting from interactions with staff delivering the trial, environmental effects due to inpatient care during placebo-controlled trials, or other unaccounted for factors, such as dietary or exercise changes during the trial [7–9]. Nonetheless, there is evidence that placebo administration results in ‘true’ - or non-artefactual - placebo effects, that is, identifiable changes in biological systems [1, 10, 11]. For example, placebo administration is capable of causing immunosuppression [12, 13], placebo effects in Parkinson’s disease are driven by striatal dopamine release [10, 14], and placebo analgesia is mediated by endogenous opioid release [15, 16]. Furthermore, there is evidence that placebo effects in depressive and anxiety disorders are correlated with altered activity in the ventral striatum, orbitofrontal cortex, rostral anterior cingulate cortex, and the default mode network [17]. The placebo effect size can be increased through the use of verbal suggestions and conditioning procedures, thus suggesting the underlying role of psychological mechanisms including learning and expectations [11, 18].

Across age groups, treatment modalities, and diverse mental disorders, biological treatments (pharmacotherapy or neurostimulation) do reduce symptoms [19–22], but only a subgroup of patients experience a clinically significant symptom response or enter remission [23–25]. Furthermore, current medications may also have unfavourable side effects [23, 26–31]. Given the high prevalence of mental disorders and their significant socioeconomic burden [32–34], there is a need to develop more effective and safer psychopharmacologic and neurostimulation treatments. However, in randomized-controlled trials (RCTs), the magnitude of the placebo response may be considerable, which can affect the interpretation of their results [35–37]. For example, in antipsychotic trials over the past 40 years, placebo response has increased while medication response has remained consistent [38, 39]. Consequently, the trial’s ability to statistically differentiate between an active medication and a placebo is diminished [40]. Indeed, large placebo response rates have been implicated in hindering psychotropic drug development [41, 42]. The increased placebo response can also affect larger data synthesis approaches, such as network meta-analysis, in which assumptions about placebo responses (e.g. stability over time) might affect the validity of results [43].

Improved understanding of participant, trial, and mental disorder-related factors that contribute to placebo response might allow better clinical trial design to separate active treatment from placebo effects. There is a growing body of research, including individual studies and systematic reviews/meta-analyses, examining the placebo response within specific mental disorders [35]. However, to date, no overarching synthesis of this literature, to detect any similarities or differences across mental disorders, has been published. We therefore carried out an umbrella review of meta-analyses to address this need. We aimed to assess the placebo effect size in RCTs for a range of mental disorders, whether the effect size differs across distinct mental disorders, and identify any correlates of increased placebo effect size or response rate.

## Methods

The protocol for this systematic umbrella review was pre-registered on the open science framework (https://osf.io/fxvn4/) and published [44]. Deviations from this protocol, and additions to it, were: eight authors were involved in record screening rather than two; we reported effect sizes pooled across age groups and analyses comparing placebo effect sizes between age groups; and we included a meta-analysis that incorporated trials of dietary supplements as well as medications in autism. For the rationale behind these decisions, see eMethods.

Eight authors (NH, AB, VB, LE, OKF, LM, CR, SS) carried out the systematic review and data extraction independently in pairs. Discrepancies were resolved through consensus or through arbitration by a third reviewer (NH or SCo). We searched, without date or language restrictions, up to 23.10.2022, Medline, PsycInfo, EMBASE + EMBASE Classic, and Web of Knowledge for systematic reviews with or without meta-analyses of RCTs of biological treatments (psychopharmacotherapy or neurostimulation) compared with a placebo or sham treatment in individuals with mental disorders diagnosed according to standardized criteria. The full search strategy is included in eMethods. We also sought systematic reviews of RCTs conducted in patients with sleep-wake disorders, since these disorders are included in the DSM-5 and their core symptoms overlap with those of mental disorders [45]. We retained systematic reviews with or without meta-analyses that reported within-group changes in symptoms in the placebo arm.

Next, to prevent duplication of data, a matrix containing all eligible systematic reviews/meta-analyses for each category of mental disorder was created. Where there were multiple eligible systematic reviews/meta-analyses for the same disorder and treatment, we preferentially included meta-analyses, and if multiple eligible meta-analyses remained, then we included the one containing the largest number of studies for the same disorder and treatment, in line with recent umbrella reviews [46, 47].

Data were extracted by at least two among six reviewers (AB, VB, LE, OKF, CR, SS) independently in pairs via a piloted form. All extracted data were further checked by a third reviewer (NH). See eMethods for a list of extracted data.

Our primary outcome was the pre-post effect size of the placebo/sham related to the condition-specific primary symptom change for each mental disorder. Secondary outcomes included any other reported clinical outcomes in eligible reviews. We report effect sizes calculated *within-group* from baseline and post-treatment means by meta-analysis authors, including Cohen’s *d* and Hedges’ *g* for repeated measures, which account for both mean difference and correlation between paired observations; and standardized mean change, where the average change score is divided by standard deviation of the change scores. We interpreted the effect size in line with the suggestion by Cohen [48], i.e. small (~0.2), medium (~0.5), or large (~0.8).

In addition, we extracted data regarding potential correlates of increased placebo effect size or response rate (as defined and assessed by the authors of each meta-analysis) in each mental disorder identified through correlation analyses or meta-regression. Where available, results from multivariate analyses were preferred.

The methodological quality of included reviews was assessed by at least two among six reviewers (AB, VB, LE, OKF, NH, CR) independently and in pairs using the AMSTAR-2 tool, a critical appraisal tool that enables reproducible assessments of the conduct of systematic reviews [49]. The methodological quality of each included review was rated as high, moderate, low, or critically low.

## Results

**Fig. 1:**
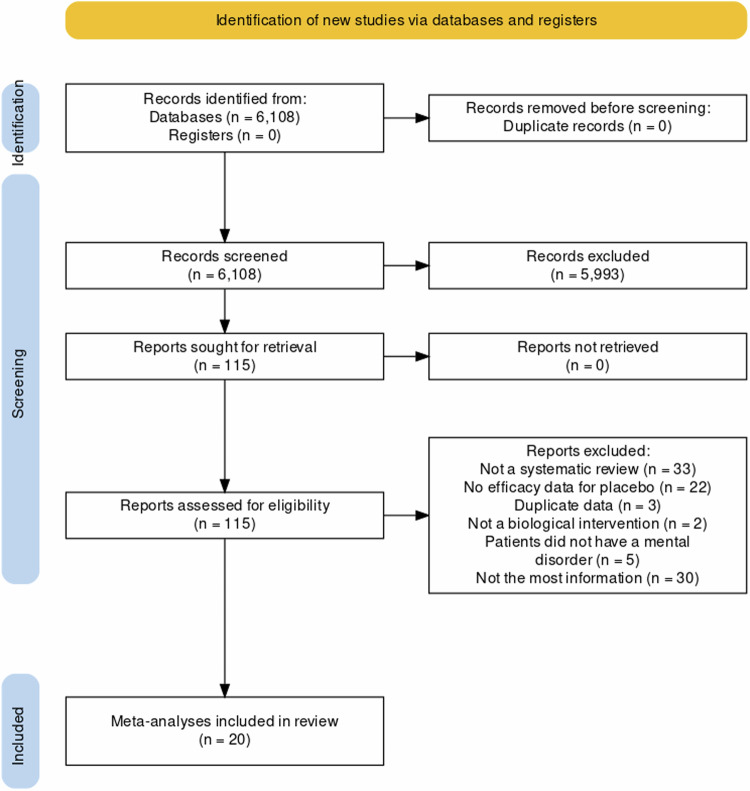
PRISMA flow diagram. Twenty meta-analyses were included.

Our initial search identified 6,108 records. After screening titles and abstracts, we obtained and assessed 115 full-text reports (see eResults for a list of articles excluded following full-text assessment, with reasons). Of these, 20 were deemed eligible, and all were systematic reviews with meta-analysis (Fig. 1). In total, the 20 included meta-analyses synthesized data from 1,691 RCTs (median 55) involving 261,730 patients (median 5,365). These meta-analyses were published between 2007 and 2022 and involved individuals with the following mental disorders: major depressive disorder (MDD; *n* = 6) [50–55], anxiety disorders (*n* = 4) [55–58], schizophrenia spectrum disorders (*n* = 3) [38, 59, 60], alcohol use disorder (AUD; *n* = 1) [61], attention-deficit/hyperactivity disorder (ADHD; *n* = 1) [62], autism spectrum disorders (*n* = 1) [63], bipolar depression (*n* = 1) [64], intellectual disability (*n* = 1) [65], obsessive-compulsive disorder (OCD; *n* = 1) [66], primary insomnia (*n* = 1) [67], and restless legs syndrome (RLS; *n* = 1) [68].

The methodological quality of the included meta-analyses according to AMSTAR-2 ratings was high in two meta-analyses (ADHD and autism), low in four meta-analyses, and critically low in the remaining 14 meta-analyses (Table 1). The most common sources of bias that led to downgrading on the AMSTAR-2 were: no list of excluded full-text articles with reasons (*k* = 14), no explicit statement that the protocol was pre-registered (*k* = 14), and no assessment of the potential impact of risk of bias in individual studies on the results (*k* = 13). The full reasoning behind our AMSTAR-2 ratings is included in eResults.

**Table 1:** Summary of included meta-analyses.

						Outcomes of Interest Reported		
Age Group or Sub-population	Reference	Mental Disorder	*k*	*N*	Mean Duration (weeks)	Placebo Effect Size	Correlates of Placebo Effect Size	Methodological Quality
*Adults*								
	Scott AJ et al., 2022 [50]	Major Depressive Disorder	347	89,183	8.92	Yes	No	Low
	Furukawa et al., 2016 [51]	Major Depressive Disorder	252	26,324	7.10	No	Yes	Low
	Leucht et al., 2018 [59]	Schizophrenia Spectrum	167	28,102	6.00	No	Yes	Low
	Agid et al., 2013 [38]	Schizophrenia Spectrum	61	14,787	6.25	Yes	Yes	Critically low
	Bandelow et al., 2015 [57]	GAD, SAD	88	20,750	10.14	Yes	Yes	Critically low
	Ahmadzad-Asl et al., 2022 [56]	Panic Disorder	43	2392	8.51	Yes	Yes	Critically low
	Silva et al., 2017 [68]	Restless Legs Syndrome	85	5046	7.87	Yes	Yes	Critically low
	Del Re et al., 2013 [61]	Alcohol Use Disorder	47	NR	NR	Yes	Yes	Critically low
	Winkler and Rief, 2015 [67]	Primary Insomnia	32	3969	4.53	Yes	No	Critically low
	Curie et al., 2015 [65]	Intellectual Disability	22	721	35.00	Yes	Yes	Critically low
	Iovieno et al., 2016 [64]	Bipolar Depression	15	7502	7.50	No	Yes	Critically low
*Children & Adolescents*								
	Locher et al., 2017 [55]	Major Depressive Disorder,						
		Anxiety Disorders	27	5840	9.89	Yes	No	Critically low
	Meister et al., 2020 [54]	Major Depressive Disorder	24	2229	10.04	No	Yes	Critically low
*Older Adults (>60 years)*								
	Pinquart et al., 2007 [58]	Anxiety Disorders	13	1273	8.00	Yes	No	Critically low
*Mixed Ages*								
	Faraone et al., 2022 [62]	ADHD	123	19,753	7.42	Yes	Yes	High
	Siafis et al., 2020 [63]	Autism Spectrum	86	5365	11.20	Yes	Yes	High
	Mohamadi et al., 2022 [66]	OCD	49	1993	13.00	Yes	Yes	Critically low
*Negative Symptoms of Schizophrenia*								
	Czobor et al., 2022 [60]	Schizophrenia Spectrum	25	4391	14.80	Yes	Yes	Critically low
*Treatment-Resistant*								
	Scott F et al., 2022 [52]	Major Depressive Disorder	115	21,172	9.00	Yes	No	Low
*Neurostimulation*								
	Razza et al., 2018 [53]	Major Depressive Disorder	61	1328	NR	Yes	Yes	Critically low

*k* number of component studies, *N* number of patients, *GAD* generalised anxiety disorder, *SAD* social anxiety disorder, *ADHD* attention-deficit hyperactivity disorder; *OCD* obsessive-compulsive disorder, *NR* not reported.

**Fig. 2:**
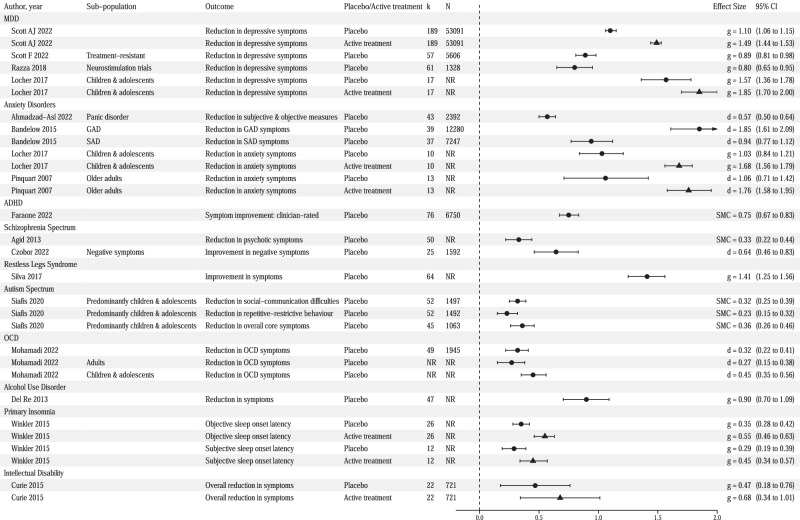
Forest plot of effect sizes for within-group change in placebo and active treatment groups. Dots represent placebo group effect size while triangles represent active effect size. CI confidence interval, MDD major depressive disorder, GAD generalized anxiety disorder, SAD social anxiety disorder, OCD obsessive-compulsive disorder, g Hedges’ g, d Cohen’s d, SMC standardized mean change, NR not reported.

Our first objective was to determine placebo effect sizes across mental conditions. Data regarding within-group placebo efficacy were reported in sixteen of the included meta-analyses [38, 50, 52, 53, 55–58, 60–63, 65–68]. Placebo effect sizes for the primary outcomes ranged from 0.23 to 1.85, with a median of 0.64 (Fig. 2). Median heterogeneity across meta-analyses was I^2^ = 72%, suggesting a generally high percentage of heterogeneity due to true variation across studies.

A detailed description of each meta-analysis included for this objective is included in eResults. Here, we report a summary of these results in order of the greatest number of RCT’s and meta-analyses included per disorder. In MDD, a large within-group placebo effect was observed (*g* = 1.10, 95% CI [1.06, 1.15]), although active medication had an even larger effect size (*g* = 1.49, 95% CI [1.44, 1.53]) [50]. Similarly, in children and adolescents with MDD, placebo effect size was large (*g* = 1.57, 95% CI [1.36, 1.78]), as was serotonergic medication effect size (*g* = 1.85, 95% CI [1.70, 2.00]) [55]. In treatment-resistant MDD, the within-group placebo effect size was smaller than in non-treatment-resistant MDD (*g* = 0.89, 95% CI [0.81, 0.98]) [52]. In neuromodulation trials for MDD, the effect size of sham was *g* = 0.80 (95% CI [0.65, 0.95]) [53]. In this meta-analysis, the effect size was larger for non-treatment-resistant (*g* = 1.28, 95% CI [0.47, 2.97]) compared to treatment-resistant participants (g = 0.50 95% CI [0.03, 0.99]) [53]. In adults with anxiety disorders, placebo effect sizes varied across disorders, with a medium effect size in panic disorder (*d* = 0.57, 95% CI [0.50, 0.64]) [56] and large effect sizes in generalized anxiety disorder (GAD) (*d* = 1.85, 95% CI [1.61, 2.09]) and social anxiety disorder (SAD) (*d* = 0.94, 95% CI [0.77, 1.12]) [57]. Other meta-analyses in children and adolescents and older adults pooled RCTs across anxiety disorders, and found large placebo effect sizes (*g* = 1.03, 95% CI [0.84, 1.21] and *d* = 1.06, 95% CI [0.71, 1.42], respectively) [55, 58]. In ADHD, placebo effect size was medium-to-large for clinician-rated outcomes (SMC = 0.75, 95% CI [0.67, 0.83]) [62]. There was additionally a significant negative relationship between placebo effect size and drug-placebo difference (−0.56, *p* < 0.01) for self-rated outcomes [62]. In schizophrenia spectrum disorders, placebo effect size was small-to-medium in antipsychotic RCTs (SMC = 0.33, 95% CI [0.22, 0.44]) [38] and medium in RCTs focusing specifically on negative symptoms (*d* = 0.64, 95% CI [0.46, 0.83]) [60]. Placebo effect size in RLS was large when measured via rating scales (*g* = 1.41, 95% CI [1.25, 1.56]), but small (*g* = 0.02 to 0.24) in RCTs using objective outcomes [68]. In autism, placebo effect sizes were small (SMC ranged 0.23 to 0.36) [63]. Similarly, placebo effect size was small in OCD (*d* = 0.32, 95% CI [0.22, 0.41]), although larger in children and adolescents (*d* = 0.45, 95% CI [0.35, 0.56]) compared with adults (*d* = 0.27, 95% CI [0.15, 0.38]) [66]. Placebo effect size was large in AUD (*g* = 0.90, 95% CI [0.70, 1.09]) [61], small in primary insomnia (*g* ranged 0.25 to 0.43) [67], and medium in intellectual disability related to genetic causes (*g* = 0.47, 95% CI [0.18, 0.76]) [65].

Our second objective was to examine the correlates of increased placebo response. We included 14 meta-analyses that reported correlates of placebo effect size or response rate through correlation analysis or meta-regression [38, 51, 53, 54, 56, 57, 59–64, 66, 68]. The key correlates extracted from these studies are summarized in Table 2.

**Table 2:** Significant correlates of placebo effect size or response rate identified either through meta-regression or correlation analyses.

	Patient Population						
Reference	Mental Disorder	Sub-population	*k*	Correlate	Relationship Measure	Statistic	95% CI
Furukawa et al., 2016 [51]	Major Depressive Disorder	Adults	112	Multicenter vs single center	RR	1·41	1·22, 1·62
				Flexible vs Fixed dose	RR	1·17	1·04, 1·31
				Trial duration (every 1 week increase)	RR	1·04	1·01, 1·06
Razza et al., 2018 [53]	Major Depressive Disorder	Neurostimulation trials	61	Active effect size	Beta	0·28	0·12, 0·43
Meister et al., 2020 [54]	Major Depressive Disorder	Children & adolescents	24	Number of sites	OR	1·01	1·00, 1·02
Ahmadzad-Asl et al., 2022 [56]	Anxiety Disorders	Panic Disorder	NR	Publication year	Beta	0·02	0·01, 0·04
				Number of participants	Beta	0·001	0·000, 0·002
Bandelow et al., 2015 [57]	Anxiety Disorders	GAD, SAD, Panic Disorder	111	Publication year	r	0·41	NR
Agid et al., 2013 [38]	Schizophrenia Spectrum	Adults	61	Publication year	Regression coefficient	0·04	NR
				Age	Regression coefficient	−0·05	NR
				Illness duration	Regression coefficient	−0·05	NR
				Baseline CGI severity	Regression coefficient	0·42	NR
				Trial duration	Regression coefficient	−0·08	NR
Leucht et al., 2018 [59]	Schizophrenia Spectrum	Adults	95	Number of participants (10 participants more)	Regression coefficient	0·15	0·07, 0·24
				Average age	Regression coefficient	−6·23	−10·46, −2·00
				Number of sites	Regression coefficient	1·38	0·71, 2·04
Czobor et al., 2022 [60]	Schizophrenia Spectrum	Negative symptoms	25	Increased number of active treatment arms	Regression coefficient	0·25	NR
				Trial duration	Regression coefficient	−0·37	−0·65, −0·08
Faraone et al., 2022 [62]	ADHD	Mixed ages	NR	Active medication effect size (clinician-rated outcomes)	r	0·69	NR
				Active medication effect size (parent-rated outcomes)	r	0·44	NR
				Active medication effect size (teacher-rated outcomes)	r	0·85	NR
Del Re et al., 2013 [61]	Alcohol Use Disorder	Adults	47	Publication year	Regression coefficient	0·04	0·00, 0·08
			36	Number of administrations	Regression coefficient	0·30	0·00, 0·61
			47	Baseline severity	Regression coefficient	0·22	0·01, 0·43
Siafis et al., 2020 [63]	Autism Spectrum	Predominantly children & adolescents	51	Active medication effect size on overall core symptoms	Spearman’s *ρ*	0·54	NR
				Active medication effect size on social-communication difficulties	Spearman’s *ρ*	0·53	NR
				Number of sites	Regression coefficient	0·03	0·01, 0·05
				Low risk of bias in allocation concealment	Regression coefficient	0·25	0·02, 0·49
Mohamadi et al., 2022 [66]	OCD	Mixed ages	NR	Publication year	Beta	0·01	NR
				Follow up duration (weeks)	Beta	−0·01	NR
				Mean age (years)	Beta	−0·01	NR
				Number of participants	Beta	0·003	NR
Iovieno et al., 2016 [64]	Bipolar Depression	Adults	15	Active medication response rate	Regression coefficient	0·892	NR
				Relative risk of responding to active medication vs placebo	Regression coefficient	−1·11	NR

Correlates are included here only if they were reported as significant. Results from meta-regressions were preferred, and results from multivariate versus univariate regressions were preferred where these were available. Positive statistics signify correlates of larger placebo response, while negative statistics signify correlates of smaller placebo response.

*k* number of component studies, *GAD* generalised anxiety disorder, *SAD* social anxiety disorder, *ADHD* attention-deficit hyperactivity disorder, *OCD* obsessive-compulsive disorder, *RR* relative risk, *OR* odds ratio, *r* Pearson’s correlation coefficient, *NR* not reported.

Several variables were consistently identified across meta-analyses. Increased number of trial sites was a positive correlate of increased placebo response in MDD [51, 54], schizophrenia spectrum disorders [59], and autism spectrum disorders [63]. Similarly, increased sample size was positively associated with placebo effect size in schizophrenia spectrum disorders [59], OCD [66], and panic disorder [56]. Later publication or study year was associated with greater placebo response in anxiety disorders [56, 57], schizophrenia spectrum disorders [38], AUD [61], and OCD [66] but not in MDD [51], and with *reduced* placebo response in ADHD [62]. Younger age was associated with increased placebo responses in schizophrenia spectrum disorders [38, 59] and OCD [66]. Increased baseline illness severity was associated with increased placebo response in schizophrenia spectrum disorders [38], ADHD [62], and AUD [61]. Increased trial or follow-up duration was positively associated with increased placebo response in MDD [51], but negatively associated with placebo response in schizophrenia spectrum disorders [38, 60] and OCD [66]. Finally, the effect size of active treatment was positively associated with increased placebo response in neurostimulation trials for MDD [53], bipolar depression [64], autistic spectrum disorders [63], and ADHD [62].

There were also some variables associated with increased placebo response in single disorders only. Flexible dosing, rather than fixed dosing, was associated with increased placebo response in MDD [51]. Increased illness duration was associated with reduced placebo response in schizophrenia spectrum disorders [38]. In RCTs for negative symptoms of schizophrenia, a higher number of active treatment arms was associated with increased placebo response [60]. A number of treatment administrations was a positive correlate of increased placebo response in patients with AUD [61]. A low risk of bias in selective reporting was associated with increased placebo response in ADHD [62]. Finally, a low risk of bias in allocation concealment was associated with increased placebo response in autism [63].

## Discussion

**Fig. 3:**
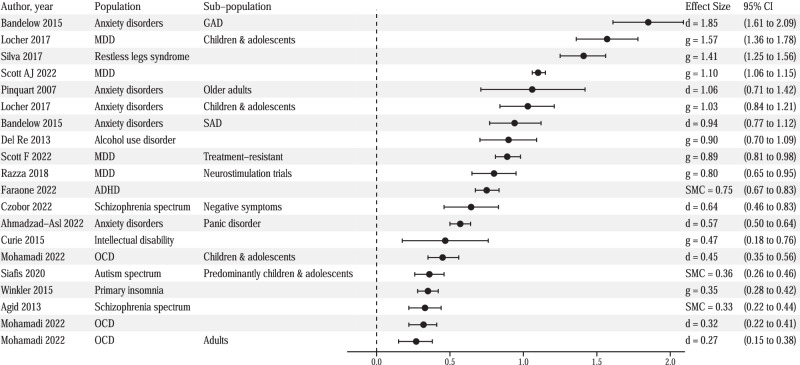
Forest plot of effect sizes for within-group change in placebo groups, ordered by magnitude. CI confidence interval, MDD major depressive disorder, GAD generalized anxiety disorder, SAD social anxiety disorder, OCD obsessive-compulsive disorder, g Hedges’ g, d Cohen’s d, SMC standardized mean change.

To our knowledge, this is the first overarching synthesis of the literature exploring the placebo response in RCTs of biological treatments across a broad range of mental disorders. We found that placebo responses were present and detectable across mental disorders. Further, the placebo effect size across these disorders varied between small and large (see Fig. 3). Additionally, several variables appeared to be associated with increased placebo effect size or response rate across a number of disorders, while others were reported for individual disorders only.

Our umbrella review distinguishes itself from a recent publication on placebo mechanisms across medical conditions [69]. Only four systematic reviews of research in mental disorders were included in that recent review [69], none of which were eligible for inclusion in our umbrella review, as we focus specifically on RCTs in mental disorders. Thus, our current umbrella review synthesizes different literature and is complementary [69].

We found substantial variation in placebo effect sizes across mental disorders. In GAD, SAD, MDD, AUD, and RLS (for subjective outcomes), placebo effects were large (>0.9), while they were small (approximately 0.3) in OCD, primary insomnia, autism, RLS (for objective outcomes), and schizophrenia spectrum disorders. It is noteworthy that placebo effect size/response rate correlated with active treatment effect size/response rate in many disorders (MDD, bipolar depression, ADHD, and autism). Nonetheless, where reported, active treatment was always superior. This possibly suggests an underlying ‘treatment responsiveness’ of these disorders that can vary in size. Perhaps, the natural history of a disorder is an important factor in ‘responsiveness’, i.e., disorders in which there is greater natural fluctuation in severity will show larger placebo (and active treatment) effect sizes. Supporting this hypothesis, increased trial duration predicted a larger placebo effect size in MDD, a disorder in which the natural course includes improvement [31, 51, 70]. Conversely, in schizophrenia spectrum disorders where improvement (particularly of negative symptoms) is less likely [71], increased trial and illness duration predicted *a smaller* placebo effect size [38, 60]. However, previous meta-analyses suggest that natural improvement, for example, measured via waiting list control, does not fully account for the placebo effect in depression and anxiety disorders [72, 73]. Statistical artifact, therefore, does not seem to fully explain the variation in effect size.

Non-specific treatment mechanisms are likely an additional source of the observed placebo effect. For example, those with treatment-resistant illness might have reduced expectations regarding treatment. This assumption is supported by the subgroup analysis reported by Razza and colleagues showing sham neuromodulation efficacy reduced as the number of previous failed antidepressant trials increased [53]. Another factor to consider is the outcome measure chosen. For example, the placebo effect size in panic disorder was smaller when calculated with objective or self-report measures compared with clinician-rated measures [56]. A similar finding was reported in ADHD trials [62]. Why placebo effect sizes would differ with clinician-rated versus self-rated scales is unclear. This might result from ‘demand characteristics’ (i.e., cues that suggest to a patient how they ‘should’ respond), or unblinding of the rater, or a combination of the two [74, 75].

Several correlates of increased placebo response were reported in included meta-analyses. These included a larger sample size, more study sites, a later publication year (but with an opposite finding for ADHD), younger age, and increased baseline illness severity. This might reflect changes in clinical trial methods over time, the potential for increased ‘noise’ in the data with larger samples or more study sites, and, more speculatively, variables associated with increased volatility in symptoms [39, 51, 76]. A more extensive discussion regarding the potential reasons these variables might correlate with, or predict, placebo response is included in the eDiscussion. Although some correlates of increased placebo response were identified, perhaps more pertinently, it is unknown whether these also predict the separation between active treatment and placebo in most mental disorders. Three included meta-analyses did show that as placebo response increases, the likelihood of drug-placebo separation decreases [38, 62, 64]. This suggests correlates of placebo effect size are also correlates of trial success or failure, but this hypothesis needs explicit testing. In addition, few of the meta-analyses we included explored whether correlates of placebo response differed from correlates of active treatment response. For example, in clinical trials for gambling disorder, response to active treatment was predicted by weeks spent in the trial and by baseline severity, while response to placebo was predicted by baseline depressive and anxiety symptoms [77]. Furthermore, there is evidence that industry sponsorship is a specific correlate of reduced drug-placebo separation in schizophrenia spectrum disorders [78]. The largest meta-analysis that we included (conducted by Scott et al. [50]) did not explore correlates of increased placebo response through meta-regression analysis; rather, it was designed specifically to assess the impact of the use of placebo run-in periods in antidepressant trials. The authors found that use of a placebo run-in was associated with reduced placebo response. However, this effect did not enhance sensitivity to detect medication efficacy versus control groups, as trials with placebo run-in periods were also associated with a reduced medication response. Similar effects of placebo run-in were seen in univariate (but not multivariable) models in ADHD, where placebo run-in reduced placebo effect size in youth, but did not affect drug vs placebo difference [62]. Further work should be undertaken to ascertain whether trial-level correlates (including the use of placebo run-in) differentially explain active treatment or placebo response and whether controlling for these can improve drug-placebo separation.

Our results should be considered in the light of several possible limitations. First, as in any umbrella review, we were limited by the quality of the meta-analyses we included. Our AMSTAR-2 ratings suggest that confidence in the conclusions of most included meta-analyses should be critically low or low. Indeed, several meta-analyses did not assess for publication bias or for bias in included RCTs. This is relevant, as the risk of bias in selective reporting was highlighted as potentially being associated with placebo effect size in ADHD [62], and might therefore be relevant in other mental disorders. Second, our results are potentially vulnerable to biases or unmeasured confounders present in the included meta-analyses. Third, we attempted to prevent overlap and duplication of information by including only the meta-analyses with the most information. This might, however, have resulted in some data not being included in our synthesis. Fourth, an exploration of the potential clinical relevance of the placebo effect sizes reported here was outside the scope of the current review but should be considered an important question for future research. Finally, the meta-analyses we included encompassed RCTs with different levels of blinding (double-blind, single-blind). Although the majority of trials were likely double-blind, it is possible that different levels of blinding could have influenced placebo effect sizes through effects on expectations. Future analyses of placebo effects and their correlates should either focus on double-blind trials or compare results across levels of blinding. Related to this, the included meta-analyses pooled phase 2 and phase 3 trials (the latter of which will usually follow positive phase 2 trials), which might result in different expectation biases. Therefore, placebo effects should be compared between phase 2 and phase 3 trials in the future.

In this umbrella review, we found placebo effect sizes varied substantially across mental disorders. The sources of this variation remain unknown and require further study. Some variables were correlates of increased placebo response across mental disorders, including larger sample size, higher number of study sites, later publication year (opposite for ADHD), younger age, and increased baseline illness severity. There was also evidence that clinician-rated outcomes were associated with larger placebo effect sizes than self-rated or objective outcomes. We additionally identified important gaps in the literature, with no eligible systematic reviews identified in stress-related disorders, eating disorders, behavioural addictions, or bipolar mania. In relation to these disorders, some analyses have been published but they have not been included in systematic reviews/meta-analyses (e.g. analyses of individual patient data pooled across RCTs in acute mania [79] or gambling disorder [77, 80]) and therefore were not eligible for inclusion here. We also focused on placebo response in RCTs of pharmacotherapies and neurostimulation interventions for mental disorders. We did not include placebo effects in psychosocial interventions, but such an analysis would also be valuable. Future studies should address these gaps in the literature and furthermore should compare findings in placebo arms with active treatment arms, both regarding treatment effect size and its correlates. Gaining additional insights into the placebo response may improve our ability to separate active treatment effects from placebo effects, thus paving the way for potentially effective new treatments for mental disorders.

## Supplementary information

PLACEBO EFFECTS IN RANDOMIZED TRIALS OF PHARMACOLOGICAL AND NEUROSTIMULATION INTERVENTIONS FOR MENTAL DISORDERS: AN UMBRELLA REVIEW SUPPLEMENTARY APPENDIX

## Data Availability

The datasets generated during and/or analysed during the current study are available in the Open Science Framework repository, https://osf.io/fxvn4/.
