# Supplementary material for: Placebo effects in randomized trials of pharmacological and neurostimulation interventions for mental disorders: An umbrella review
Source: Mol Psychiatry. 2024 Jun 24;29(12):3915–25. doi: 10.1038/s41380-024-02638-x (PMC11609099; doi:10.1038/s41380-024-02638-x)
Supplement: Supplementary file 1 — PLACEBO EFFECTS IN RANDOMIZED TRIALS OF PHARMACOLOGICAL AND NEUROSTIMULATION INTERVENTIONS FOR MENTAL DISORDERS: AN UMBRELLA REVIEW SUPPLEMENTARY APPENDIX [file 41380_2024_2638_MOESM1_ESM.docx]

**PLACEBO EFFECTS IN RANDOMIZED TRIALS OF PHARMACOLOGICAL AND NEUROSTIMULATION INTERVENTIONS FOR MENTAL DISORDERS: AN UMBRELLA REVIEW**

**SUPPLEMENTARY APPENDIX**

Nathan TM Huneke, Jay Amin, David S Baldwin, Alessio Bellato, Valerie Brandt, Samuel R Chamberlain, Christoph U Correll, Luis Eudave, Matthew Garner, Corentin J Gosling, Catherine M Hill, Ruihua Hou, Oliver D Howes, Konstantinos Ioannidis, Ole Köhler-Forsberg, Lucia Marzulli, Claire Reed, Julia MA Sinclair, Satneet Singh, Marco Solmi*, Samuele Cortese*

*Contributed equally

Table of Contents

[1. eMethods 2](#_Toc153528528)

[1.1. Search Terms 2](#_Toc153528529)

[1.2. Data Extracted From Included Meta-Analyses 6](#_Toc153528530)

[1.3. Definition of Systematic Review 6](#_Toc153528531)

[1.4. Deviations from Pre-Registered Protocol 7](#_Toc153528532)

[2. eResults 8](#_Toc153528533)

[2.1. List of References Excluded after Reading Article in Full, With Reasons 8](#_Toc153528534)

[2.2. Narrative Description of Meta-Analyses Reporting Placebo Effect Size 15](#_Toc153528535)

[2.2.1. Major Depressive Disorder 15](#_Toc153528536)

[2.2.2. Anxiety Disorders 16](#_Toc153528537)

[2.2.3. Attention-Deficit/Hyperactivity Disorder 17](#_Toc153528538)

[2.2.4. Schizophrenia Spectrum Disorders 17](#_Toc153528539)

[2.2.5. Restless Legs Syndrome 18](#_Toc153528540)

[2.2.6. Autism Spectrum Disorders 18](#_Toc153528541)

[2.2.7. Obsessive-Compulsive Disorder 18](#_Toc153528542)

[2.2.8. Alcohol Use Disorder 19](#_Toc153528543)

[2.2.9. Primary Insomnia 19](#_Toc153528544)

[2.2.10. Intellectual Disability 19](#_Toc153528545)

[2.3. AMSTAR-2 Ratings 21](#_Toc153528546)

[3. eDiscussion 23](#_Toc153528547)

[4. eReferences 25](#_Toc153528548)

# 1. eMethods

## Search Terms

("attention deficit disorder with hyperactivity"[MeSH Terms] OR ("attention"[All Fields] AND "deficit"[All Fields] AND "disorder"[All Fields] AND "hyperactivity"[All Fields]) OR "attention deficit disorder with hyperactivity"[All Fields] OR "adhd"[All Fields]) OR ("attention deficit disorder with hyperactivity"[MeSH Terms] OR ("attention"[All Fields] AND "deficit"[All Fields] AND "disorder"[All Fields] AND "hyperactivity"[All Fields]) OR "attention deficit disorder with hyperactivity"[All Fields] OR ("attention"[All Fields] AND "deficit"[All Fields] AND "hyperactivity"[All Fields] AND "disorder"[All Fields]) OR "attention deficit hyperactivity disorder"[All Fields]) OR ("attention deficit disorder with hyperactivity"[MeSH Terms] OR ("attention"[All Fields] AND "deficit"[All Fields] AND "disorder"[All Fields] AND "hyperactivity"[All Fields]) OR "attention deficit disorder with hyperactivity"[All Fields] OR ("attention"[All Fields] AND "deficit"[All Fields]) OR "attention deficit"[All Fields]) OR ("attention deficit disorder with hyperactivity"[MeSH Terms] OR ("attention"[All Fields] AND "deficit"[All Fields] AND "disorder"[All Fields] AND "hyperactivity"[All Fields]) OR "attention deficit disorder with hyperactivity"[All Fields] OR ("hyperkinetic"[All Fields] AND "disorder"[All Fields]) OR "hyperkinetic disorder"[All Fields]) OR ("attention deficit disorder with hyperactivity"[MeSH Terms] OR ("attention"[All Fields] AND "deficit"[All Fields] AND "disorder"[All Fields] AND "hyperactivity"[All Fields]) OR "attention deficit disorder with hyperactivity"[All Fields] OR ("hyperkinetic"[All Fields] AND "syndrome"[All Fields]) OR "hyperkinetic syndrome"[All Fields]) OR ("autistic disorder"[MeSH Terms] OR ("autistic"[All Fields] AND "disorder"[All Fields]) OR "autistic disorder"[All Fields] OR "autism"[All Fields]) OR ("autistic disorder"[MeSH Terms] OR ("autistic"[All Fields] AND "disorder"[All Fields]) OR "autistic disorder"[All Fields] OR "autistic"[All Fields]) OR Asperger[All Fields] OR ("schizophrenia"[MeSH Terms] OR "schizophrenia"[All Fields]) OR schizophrenic[All Fields] OR ("psychotic disorders"[MeSH Terms] OR ("psychotic"[All Fields] AND "disorders"[All Fields]) OR "psychotic disorders"[All Fields] OR ("psychotic"[All Fields] AND "disorder"[All Fields]) OR "psychotic disorder"[All Fields]) OR ("psychotic disorders"[MeSH Terms] OR ("psychotic"[All Fields] AND "disorders"[All Fields]) OR "psychotic disorders"[All Fields]) OR ("psychotic disorders"[MeSH Terms] OR ("psychotic"[All Fields] AND "disorders"[All Fields]) OR "psychotic disorders"[All Fields] OR "psychosis"[All Fields]) OR ("bipolar disorder"[MeSH Terms] OR ("bipolar"[All Fields] AND "disorder"[All Fields]) OR "bipolar disorder"[All Fields]) OR ("bipolar disorder"[MeSH Terms] OR ("bipolar"[All Fields] AND "disorder"[All Fields]) OR "bipolar disorder"[All Fields] OR ("bipolar"[All Fields] AND "disorders"[All Fields]) OR "bipolar disorders"[All Fields]) OR ("depressive disorder"[MeSH Terms] OR ("depressive"[All Fields] AND "disorder"[All Fields]) OR "depressive disorder"[All Fields] OR "depression"[All Fields] OR "depression"[MeSH Terms]) OR depressive[All Fields] OR ("affect"[MeSH Terms] OR "affect"[All Fields] OR "mood"[All Fields]) OR ("anxiety"[MeSH Terms] OR "anxiety"[All Fields]) OR ("anxiety"[MeSH Terms] OR "anxiety"[All Fields] OR "anxious"[All Fields]) OR ("obsessive-compulsive disorder"[MeSH Terms] OR ("obsessive-compulsive"[All Fields] AND "disorder"[All Fields]) OR "obsessive-compulsive disorder"[All Fields] OR ("obsessive"[All Fields] AND "compulsive"[All Fields] AND "disorder"[All Fields]) OR "obsessive compulsive disorder"[All Fields]) OR ("obsessive-compulsive disorder"[MeSH Terms] OR ("obsessive-compulsive"[All Fields] AND "disorder"[All Fields]) OR "obsessive-compulsive disorder"[All Fields] OR ("obsessive"[All Fields] AND "compulsive"[All Fields] AND "disorder"[All Fields]) OR "obsessive compulsive disorder"[All Fields]) OR OCD[All Fields] OR ("stress disorders, post-traumatic"[MeSH Terms] OR ("stress"[All Fields] AND "disorders"[All Fields] AND "post-traumatic"[All Fields]) OR "post-traumatic stress disorders"[All Fields] OR "ptsd"[All Fields]) OR (Trauma-related[All Fields] AND ("disease"[MeSH Terms] OR "disease"[All Fields] OR "disorder"[All Fields])) OR (Trauma-related[All Fields] AND ("disease"[MeSH Terms] OR "disease"[All Fields] OR "disorders"[All Fields])) OR (Stress-related[All Fields] AND ("disease"[MeSH Terms] OR "disease"[All Fields] OR "disorder"[All Fields])) OR (Stress-related[All Fields] AND ("disease"[MeSH Terms] OR "disease"[All Fields] OR "disorders"[All Fields])) OR ("substance-related disorders"[MeSH Terms] OR ("substance-related"[All Fields] AND "disorders"[All Fields]) OR "substance-related disorders"[All Fields] OR ("substance"[All Fields] AND "related"[All Fields] AND "disorder"[All Fields]) OR "substance related disorder"[All Fields]) OR ("substance-related disorders"[MeSH Terms] OR ("substance-related"[All Fields] AND "disorders"[All Fields]) OR "substance-related disorders"[All Fields] OR ("substance"[All Fields] AND "related"[All Fields] AND "disorders"[All Fields]) OR "substance related disorders"[All Fields]) OR (addictive[All Fields] AND ("disease"[MeSH Terms] OR "disease"[All Fields] OR "disorder"[All Fields])) OR ("substance-related disorders"[MeSH Terms] OR ("substance-related"[All Fields] AND "disorders"[All Fields]) OR "substance-related disorders"[All Fields] OR ("substance"[All Fields] AND "disorder"[All Fields]) OR "substance use disorder"[All Fields]) OR ("substance-related disorders"[MeSH Terms] OR ("substance-related"[All Fields] AND "disorders"[All Fields]) OR "substance-related disorders"[All Fields] OR ("substance"[All Fields] AND "disorders"[All Fields]) OR "substance use disorders"[All Fields]) OR ("substance-related disorders"[MeSH Terms] OR ("substance-related"[All Fields] AND "disorders"[All Fields]) OR "substance-related disorders"[All Fields] OR ("substance"[All Fields] AND "abuse"[All Fields] AND "disorder"[All Fields]) OR "substance abuse disorder"[All Fields]) OR (("substance-related disorders"[MeSH Terms] OR ("substance-related"[All Fields] AND "disorders"[All Fields]) OR "substance-related disorders"[All Fields] OR ("substance"[All Fields] AND "abuse"[All Fields]) OR "substance abuse"[All Fields]) AND ("disease"[MeSH Terms] OR "disease"[All Fields] OR "disorders"[All Fields])) OR ("Dipend Patologiche"[Journal] OR ("addiction"[All Fields] AND "disorders"[All Fields]) OR "addiction disorders"[All Fields]) OR ("substance-related disorders"[MeSH Terms] OR ("substance-related"[All Fields] AND "disorders"[All Fields]) OR "substance-related disorders"[All Fields] OR ("drug"[All Fields] AND "abuse"[All Fields]) OR "drug abuse"[All Fields]) OR ("feeding and eating disorders"[MeSH Terms] OR ("feeding"[All Fields] AND "eating"[All Fields] AND "disorders"[All Fields]) OR "feeding and eating disorders"[All Fields] OR ("eating"[All Fields] AND "disorder"[All Fields]) OR "eating disorder"[All Fields]) OR ("feeding and eating disorders"[MeSH Terms] OR ("feeding"[All Fields] AND "eating"[All Fields] AND "disorders"[All Fields]) OR "feeding and eating disorders"[All Fields] OR ("eating"[All Fields] AND "disorders"[All Fields]) OR "eating disorders"[All Fields]) OR ("anorexia"[MeSH Terms] OR "anorexia"[All Fields]) OR ("bulimia"[MeSH Terms] OR "bulimia"[All Fields]) OR ("bulimia"[MeSH Terms] OR "bulimia"[All Fields] OR ("binge"[All Fields] AND "eating"[All Fields]) OR "binge eating"[All Fields]) OR ("bulimia"[MeSH Terms] OR "bulimia"[All Fields] OR ("binge"[All Fields] AND "eating"[All Fields]) OR "binge eating"[All Fields]) OR ("sleep wake disorders"[MeSH Terms] OR ("sleep"[All Fields] AND "wake"[All Fields] AND "disorders"[All Fields]) OR "sleep wake disorders"[All Fields] OR ("sleep"[All Fields] AND "disorder"[All Fields]) OR "sleep disorder"[All Fields]) OR ("sleep wake disorders"[MeSH Terms] OR ("sleep"[All Fields] AND "wake"[All Fields] AND "disorders"[All Fields]) OR "sleep wake disorders"[All Fields] OR ("sleep"[All Fields] AND "disorders"[All Fields]) OR "sleep disorders"[All Fields]) OR ("sleep wake disorders"[MeSH Terms] OR ("sleep"[All Fields] AND "wake"[All Fields] AND "disorders"[All Fields]) OR "sleep wake disorders"[All Fields] OR ("sleep"[All Fields] AND "disturbance"[All Fields]) OR "sleep disturbance"[All Fields]) OR ("sleep wake disorders"[MeSH Terms] OR ("sleep"[All Fields] AND "wake"[All Fields] AND "disorders"[All Fields]) OR "sleep wake disorders"[All Fields] OR ("sleep"[All Fields] AND "disturbances"[All Fields]) OR "sleep disturbances"[All Fields]) OR ("sleep initiation and maintenance disorders"[MeSH Terms] OR ("sleep"[All Fields] AND "initiation"[All Fields] AND "maintenance"[All Fields] AND "disorders"[All Fields]) OR "sleep initiation and maintenance disorders"[All Fields] OR "insomnia"[All Fields]) OR ("personality disorders"[MeSH Terms] OR ("personality"[All Fields] AND "disorders"[All Fields]) OR "personality disorders"[All Fields] OR ("personality"[All Fields] AND "disorder"[All Fields]) OR "personality disorder"[All Fields]) OR ("personality disorders"[MeSH Terms] OR ("personality"[All Fields] AND "disorders"[All Fields]) OR "personality disorders"[All Fields]) OR ("mental disorders"[MeSH Terms] OR ("mental"[All Fields] AND "disorders"[All Fields]) OR "mental disorders"[All Fields] OR ("mental"[All Fields] AND "disorder"[All Fields]) OR "mental disorder"[All Fields]) OR ("mental disorders"[MeSH Terms] OR ("mental"[All Fields] AND "disorders"[All Fields]) OR "mental disorders"[All Fields]) OR ("mental disorders"[MeSH Terms] OR ("mental"[All Fields] AND "disorders"[All Fields]) OR "mental disorders"[All Fields] OR ("psychiatric"[All Fields] AND "disorder"[All Fields]) OR "psychiatric disorder"[All Fields]) OR ("mental disorders"[MeSH Terms] OR ("mental"[All Fields] AND "disorders"[All Fields]) OR "mental disorders"[All Fields] OR ("psychiatric"[All Fields] AND "disorders"[All Fields]) OR "psychiatric disorders"[All Fields]) OR (psychopathological[All Fields] AND ("disease"[MeSH Terms] OR "disease"[All Fields] OR "disorder"[All Fields])) OR (psychopathological [All Fields] AND ("disease"[MeSH Terms] OR "disease"[All Fields] OR "disorders"[All Fields])) OR (psychopathological[All Fields] AND ("disease"[MeSH Terms] OR "disease"[All Fields] OR "condition"[All Fields])) OR (psychopathological[All Fields] AND conditions[All Fields]) OR (“neurodevelopmental disorder*”[All Fields] OR “neurodevelopmental disorder*"[MeSH Terms]) OR (“Intellectual Disabilit*”[All Fields] OR “Intellectual Disabilit*”[MeSH Terms]) OR (“mental retardation” [All Fields] OR “mental retardation” [MeSH Terms]) OR (“learning disability” [All Fields] OR “learning disability” [MeSH Terms]) OR (Schizotypal [All Fields] OR Schizotypal [MeSH Terms]) OR (“Delusional Disorder” [All Fields] OR “Delusional Disorder” [MeSH Terms]) OR (“Brief Psychotic Disorder” [All Fields] OR “Brief Psychotic Disorder”[MeSH Terms]) OR (“Schizophreniform Disorder” [All Fields] OR “Schizophreniform Disorder” [MeSH Terms]) OR (“Schizoaffective disorder” [All Fields] OR “Schizoaffective disorder” [MeSH Terms]) OR (“panic disorder” [All Fields] OR “panic disorder” [MeSH Terms]) OR (agoraphobia [All Fields] OR agoraphobia [MeSH Terms]) OR (“Body Dysmorphic Disorder” [All Fields] OR (“Body Dysmorphic Disorder” [MeSH Terms]) OR (Trichotillomania [All Fields] OR Trichotillomania [MeSH Terms]) OR (“Hair-Pulling Disorder” [All Fields] OR “Hair-Pulling Disorder” [MeSH Terms]) OR (“Excoriation Disorder” [All Fields]) OR “Excoriation Disorder” [MeSH Terms]) OR (“Skin-Picking disorder” [All Fields] OR (“Skin-Picking disorder” [MeSH Terms]) OR (“dissociative disorder*”[All Fields] OR “dissociative disorder*” [MeSH Terms]) OR (“Dissociative Identity Disorder” [All Fields] OR “Dissociative Identity Disorder” [MeSH Terms]) OR (“Restless Legs Syndrome” [All Fields] OR “Restless Legs Syndrome” [MeSH Terms]) OR (“Gender Dysphoria” [All Fields] OR “Gender Dysphoria” [MeSH Terms]) OR (“Antisocial Personality Disorder” [All Fields] OR “Antisocial Personality Disorder” [MeSH Terms]) OR (“Paranoid Personality Disorder” [All Fields] OR “Paranoid Personality Disorder” [MeSH Terms]) OR (“Schizoid Personality Disorder” [All Fields] OR “Schizoid Personality Disorder” [MeSH Terms]) OR (“Schizotypal Personality Disorder” [All Fields] OR “Schizotypal Personality Disorder” [MeSH Terms]) OR (“Borderline Personality Disorder” [All Fields] OR “Borderline Personality Disorder” [MeSH Terms]) OR (“Histrionic Personality Disorder” [All Fields] OR “Histrionic Personality Disorder” [MeSH Terms]) OR (“Narcissistic Personality Disorder” [All Fields] OR “Narcissistic Personality Disorder” [MeSH Terms]) OR (“Avoidant Personality Disorder” [All Fields] OR “Avoidant Personality Disorder” [MeSH Terms]) OR (“Dependent Personality Disorder” [All Fields] OR “Dependent Personality Disorder” [MeSH Terms]) OR (“Obsessive-Compulsive Personality Disorder” [All Fields] OR “Obsessive-Compulsive Personality Disorder” [MeSH Terms]) OR (paraphili* [All Fields] OR paraphili*[MeSH Terms]) OR (“Voyeuristic Disorder” [All Fields] OR “Voyeuristic Disorder” [MeSH Terms]) OR (“Exhibitionistic Disorder” [All Fields] OR “Exhibitionistic Disorder” [MeSH Terms]) OR (“Frotteuristic Disorder” [All Fields] OR “Frotteuristic Disorder” [MeSH Terms]) OR (“Sexual Masochism Disorder” [All Fields] OR “Sexual Masochism Disorder” [MeSH Terms]) OR (“Sexual Sadism Disorder” [All Fields] OR “Sexual Sadism Disorder” [MeSH Terms]) OR (“Pedophilic Disorder” [All Fields] OR “Pedophilic Disorder” [MeSH Terms]) OR (“Fetishistic Disorder” [All Fields] OR “Fetishistic Disorder” [MeSH Terms]) OR (“Transvestic Disorder” [All Fields] OR “Transvestic Disorder” [MeSH Terms])) AND (("systematic review"[Publication Type] OR "systematic reviews as topic"[MeSH Terms] OR "systematic review"[All Fields]) OR ("systematic review"[Publication Type] OR "systematic reviews as topic"[MeSH Terms] OR "systematic reviews"[All Fields]) OR ("meta-analysis"[Publication Type] OR "meta-analysis as topic"[MeSH Terms] OR "meta-analysis"[All Fields]) OR meta-analytic[All Fields] OR ("meta-analysis as topic"[MeSH Terms] OR ("meta-analysis"[All Fields] AND "topic"[All Fields]) OR "meta-analysis as topic"[All Fields] OR "metaanalysis"[All Fields]) OR ("meta-analysis"[Publication Type] OR "meta-analysis as topic"[MeSH Terms] OR "meta-analysis"[All Fields]) OR metanalytic[All Fields] OR metanalyses[All Fields] OR ("meta-analysis"[Publication Type] OR "meta-analysis as topic"[MeSH Terms] OR "meta-analyses"[All Fields]) OR ("meta-analysis"[Publication Type] OR "meta-analysis as topic"[MeSH Terms] OR "meta-analyses"[All Fields]) OR metaanalyses[All Fields] OR meta-review[All Fields] OR meta-reviews[All Fields] OR (umbrella[All Fields] AND ("review"[Publication Type] OR "review literature as topic"[MeSH Terms] OR "review"[All Fields])) OR (umbrella[All Fields] AND ("review"[Publication Type] OR "review literature as topic"[MeSH Terms] OR "reviews"[All Fields]))) AND (placebo [tiab] OR Nocebo [tiab])

## Data Extracted From Included Meta-Analyses

1. Author
2. Year
3. Title
4. Diagnosis
5. Disease stage/severity indicator (e.g. treatment-resistant)
6. Diagnostic criteria
7. Presence of comorbidity
8. Number of studies
9. Total sample size
10. Mean age and standard deviation
11. Mean % female and standard deviation
12. Databases searched by authors
13. Inclusion of unpublished data (yes/no)
14. Sensitivity or subgroup analyses
15. Placebo type (pill, sham, etc.)
16. Test for publication bias or small study effects (yes/no)
17. Quality appraisal (yes/no)
18. Quality appraisal tool
19. Meta-regression performed (yes/no)
20. Number of studies included in meta-regression
21. Predictors of placebo response
22. Measure of relationship (beta, r, RR, etc.)
23. Test statistic for meta-regression predictor and 95% CI
24. Primary outcome
25. Number of studies for primary outcome
26. Sample size for primary outcome
27. Placebo effect size for primary outcome and 95% CI
28. Heterogeneity for placebo for primary outcome
29. Active effect size for primary outcome and 95% CI
30. Heterogeneity for active for primary outcome
31. Active vs placebo effect size and 95% CI for primary outcome
32. Heterogeneity for active vs placebo for primary outcome
33. Secondary outcomes
34. Number of studies for each secondary outcome
35. Placebo effect size for each secondary outcome and 95% CI
36. Heterogeneity for placebo for each secondary outcome
37. Active effect size for each secondary outcome and 95% CI
38. Heterogeneity for active for each secondary outcome
39. Active vs placebo effect size for each secondary outcome and 95% CI
40. Heterogeneity for active vs placebo for each secondary outcome

## Definition of Systematic Review

We defined a systematic review in line with recent recommendations^1^, i.e., a review in which: 1) specific research question(s) were presented; 2) at least two sources were searched with a reproducible strategy; 3) inclusion and exclusion criteria were defined; 4) study selection methods were specified; and 5) a list of included studies was reported.

## Deviations from Pre-Registered Protocol

In our original protocol, we stated that two authors would screen titles, abstracts and full texts. In the final study, eight authors were involved in this process (NH, AB, VB, LE, OKF, LM, CR, SS).

We originally intended to synthesise data separately for children and adolescents (aged ≤18 years) and for patients aged >18 years where data co-occurred in a single meta-analysis in these age groups. However, in some cases this was not possible, as summary statistics for included RCTs were not reported. Instead, we report pooled effect sizes across these age groups and report results of analyses where placebo effect sizes were compared between children or adolescents and adults.

Third, our search identified only two potentially eligible meta-analyses in patients with autism spectrum disorders, both of which also included a small number of RCTs of dietary supplements.^2,3^ Although dietary supplements are not medication *per se,* we elected to include the meta-analysis of these two with the most studies to ensure we included as broad a selection of mental disorders as possible.

# 2. eResults

## 2.1. List of References Excluded after Reading Article in Full, With Reasons

**Key:** Reference
 *Reason for exclusion*

Ackerman DL, Greenland S. Multivariate meta-analysis of controlled drug studies for obsessive-compulsive disorder. *J Clin Psychopharmacol* 2002;22(3):309-17.

*Reason: Not the most information*

Ascher-Svanum H, Stensland MD, Kinon BJ, et al. Weight gain as a prognostic indicator of therapeutic improvement during acute treatment of schizophrenia with placebo or active antipsychotic. 2005.

*Reason: Not a systematic review*

Balkom AJv, Bakker A, Spinhoven P, et al. A meta-analysis of the treatment of panic disorder with or without agoraphobia: a comparison of psychopharmacological, cognitive-behavioral, and combination treatments. 1997.

*Reason: No efficacy data for placebo*

Bartoli F, Clerici M, Brita CD, et al. Effect of clinical response to active drugs and placebo on antipsychotics and mood stabilizers relative efficacy for bipolar depression and mania: A meta-regression analysis. *J Psychopharmacol* 2018;32(4):416-22.

*Reason: Not a systematic review*

Breilmann J, Furukawa TA, Becker T, et al. Differences in the placebo response in duloxetine and venlafaxine trials. *Acta Psychiatr Scand* 2018;137(6):472-80.

*Reason: Not the most information*

Brunoni AR, Lopes M, Kaptchuk TJ, et al. Placebo response of non-pharmacological and pharmacological trials in major depression: a systematic review and meta-analysis. *PloS one* 2009;4(3):e4824.

*Reason: Not the most information*

Burke MJ, Romanella SM, Mencarelli L, et al. Placebo effects and neuromodulation for depression: a meta-analysis and evaluation of shared mechanisms. *Mol Psychiatry* 2022;27(3):1658-66.

*Reason: No efficacy data for placebo*

Cao B, Liu YS, Selvitella A, et al. Differential power of placebo across major psychiatric disorders: a preliminary meta-analysis and machine learning study. Scientific reports 2021;11(1):21301.

*Reason: Not a systematic review*

Castells X, Saez M, Barcheni M, et al. Placebo Response and Its Predictors in Attention Deficit Hyperactivity Disorder: A Meta-Analysis and Comparison of Meta-Regression and MetaForest. *Int J Neuropsychopharmacol* 2022;25(1):26-35.

*Reason: Not the most information*

Cohen D, Deniau E, Maturana A, et al. Are child and adolescent responses to placebo higher in major depression than in anxiety disorders? A systematic review of placebo-controlled trials. PloS one 2008;3(7):e2632.

*Reason: Not a systematic review*

Cuestas ME, Cuestas E. [Approximate entropy of the placebo effect in clinical trials of newer antidepressants]. *Rev Fac Cien Med Univ Nac Cordoba* 2010;67(4):141-9.

*Reason: Not a systematic review*

Dollfus S, Lecardeur L, Morello R, et al. Placebo Response in Repetitive Transcranial Magnetic Stimulation Trials of Treatment of Auditory Hallucinations in Schizophrenia: A Meta-Analysis. *Schizophr Bull* 2016;42(2):301-8.

*Reason: Not a systematic review*

Fernández-López R, Riquelme-Gallego B, Bueno-Cavanillas A, et al. Influence of placebo effect in mental disorders research: A systematic review and meta-analysis. *Eur J Clin Invest* 2022;52(7):e13762.

*Reason: Not the most information and includes sham psychotherapy*

Ferreira JJ, Trenkwalder C, Mestre TA. Placebo and nocebo responses in other movement disorders besides Parkinson's disease: How much do we know? 2018.

*Reason: Not a systematic review*

Fraguas D, Díaz-Caneja CM, Pina-Camacho L, et al. Predictors of placebo response in pharmacological clinical trials of negative symptoms in schizophrenia: a meta-regression analysis. *Schizophrenia Bulletin* 2019;45(1):57-68.

*Reason: Not the most information*

Fulda S, Wetter TC. Where dopamine meets opioids: a meta-analysis of the placebo effect in restless legs syndrome treatment studies. *Brain* 2008;131(Pt 4):902-17.

*Reason: Not the most information*

Goldberg SB, Pace BT, Nicholas CR, et al. The experimental effects of psilocybin on symptoms of anxiety and depression: A meta-analysis. *Psychiatry Res* 2020;284:112749.

*Reason: Not a systematic review*

Grant JE, Chamberlain SR. The placebo effect and its clinical associations in gambling disorder. *Annals of clinical psychiatry: official journal of the American Academy of Clinical Psychiatrists* 2017;29(3):167.

*Reason: Not a systematic review*

Hamza T, Furukawa TA, Orsini N, et al. A dose-effect network meta-analysis model with application in antidepressants using restricted cubic splines. *Stat Methods Med Res* 2022:9622802211070256.

*Reason: No efficacy data for placebo*

He D, Jiang B, Guo Z, et al. Biphasic feature of placebo response in primary insomnia: pooled analysis of data from randomized controlled clinical trials of orexin receptor antagonists. *Sleep* 2020;43(3).

*Reason: Not the most information*

Holper L. Raising Placebo Efficacy in Antidepressant Trials Across Decades Explained by Small-Study Effects: A Meta-Reanalysis. *Front Psychiatry* 2020;11:633.

*Reason: Duplicate data*

Holper L, Hengartner MP. Comparative efficacy of placebos in short-term antidepressant trials for major depression: a secondary meta-analysis of placebo-controlled trials. *BMC Psychiatry* 2020;20(1):437.

*Reason: No pooled placebo effect size*

Horder J, Matthews P, Waldmann R. Placebo, prozac and PLoS: significant lessons for psychopharmacology. *J Psychopharmacol* 2011;25(10):1277-88.

*Reason: Not a systematic review*

Hougaard E. [The relationship between the placebo effect and spontaneous improvement in research on antidepressants. Are placebos powerless?]. 2005.

*Reason: Not a systematic review*

Hougaard E, Nielsen T, Zachariae B. [Placebo effect in the drug therapy of depression]. *Ugeskr Laeger* 2000;162(16):2314-7.

*Reason: Not a systematic review*

Hróbjartsson A, Gøtzsche PC. Placebo treatment versus no treatment. 2003.

*Reason: Patients did not have a mental disorder*

Hróbjartsson A, Gøtzsche PC. Placebo interventions for all clinical conditions. 2004.

*Reason: Not the most information*

Hróbjartsson A, Gøtzsche PC. Is the placebo powerless? Update of a systematic review with 52 new randomized trials comparing placebo with no treatment. 2004.

*Reason: Duplicate data*

Hróbjartsson A, Gøtzsche PC. Placebo interventions for all clinical conditions. 2010.

*Reason: No pooled effect size for placebo in mental disorders*

Hyde AJ, May BH, Xue CC, et al. Variation in Placebo Effect Sizes in Clinical Trials of Oral Interventions for Management of the Behavioral and Psychological Symptoms of Dementia (BPSD): A Systematic Review and Meta-Analysis. *Am J Geriatr Psychiatry* 2017;25(9):994-1008.

*Reason: Not a systematic review*

Jensen JS, Bielefeldt A, Hróbjartsson A. Active placebo control groups of pharmacological interventions were rarely used but merited serious consideration: a methodological overview. *Journal of Clinical Epidemiology* 2017;87:35-46.

*Reason: No efficacy data for placebo*

Jiang B, He D, Gao Z. Efficacy and Placebo Response of Multimodal Treatments for Primary Insomnia: A Network Meta-Analysis. *Clin Neuropharmacol* 2019;42(6):197-202.

*Reason: Not the most information*

Jiang B, He D, Guo Z, et al. Dynamic features of placebo effects addressing persistent insomnia disorder: A meta-analysis of placebo-controlled randomized clinical trials. *J Sleep Res* 2020;29(4):e12997.

*Reason: Includes sham psychotherapy*

Jones BDM, Razza LB, Weissman CR, et al. Magnitude of the Placebo Response Across Treatment Modalities Used for Treatment-Resistant Depression in Adults: A Systematic Review and Meta-analysis. *JAMA Netw Open* 2021;4(9):e2125531.

*Reason: Not the most information*

Khan A, Detke M, Khan SR, et al. Placebo response and antidepressant clinical trial outcome. *J Nerv Ment Dis* 2003;191(4):211-8.

*Reason: Not a systematic review*

Kingsberg S, Goldstein I, Kim NN, et al. Female Sexual Dysfunction and the Placebo Effect: A Meta-analysis. *Obstet Gynecol* 2018;132(6):1504.

*Reason: Not a systematic review*

Kirsch I. Placebo Effect in the Treatment of Depression and Anxiety. *Front Psychiatry* 2019;10:407.

*Reason: Not a systematic review*

Kirsch I. "Re: The Impact of Placebo Response Rates on Clinical Trial Outcome: A Systematic Review and Meta-Analysis of Antidepressants in Children and Adolescents with Major Depressive Disorder"" by Li Y, Huang J, He Y, Yang J, Lv Y, Liu H, Liang L, Zheng Q, and Li L (J Child Adolesc Psychopharmacol 29:712-720, 2019)""". 2020.

*Reason: Not a systematic review*

Koponen H, Lepola U. Psychiatric drug trials and placebo. *Nord J Psychiatry* 2005;59(4):233-7.

*Reason: Not a systematic review*

Krogsbøll LT, Hróbjartsson A, Gøtzsche PC. Spontaneous improvement in randomised clinical trials: meta-analysis of three-armed trials comparing no treatment, placebo and active intervention. 2009.

*Reason: Unclear intervention*

Krol FJ, Hagin M, Vieta E, et al. Placebo-To be or not to be? Are there really alternatives to placebo-controlled trials? 2020.

*Reason: No efficacy data for placebo*

Kumagai F, Suzuki T, Fleischhacker WW, et al. Early Placebo Improvement Is a Marker for Subsequent Placebo Response in Long-Acting Injectable Antipsychotic Trials for Schizophrenia: Combined Analysis of 4 RCTs. 2018.

*Reason: Not a systematic review*

Lee S, Walker JR, Jakul L, et al. Does elimination of placebo responders in a placebo run-in increase the treatment effect in randomized clinical trials? A meta-analytic evaluation. 2004.

*Reason: No efficacy data for placebo*

Leucht S, Chaimani A, Mavridis D, et al. Disconnection of drug-response and placebo-response in acute-phase antipsychotic drug trials on schizophrenia? Meta-regression analysis. *Neuropsychopharmacology* 2019;44(11):1955-66.

*Reason: Duplicate data*

Li F, Nasir M, Olten B, et al. Meta-analysis of placebo response in adult antidepressant trials. CNS drugs 2019;33:971-80.

*Reason: Not a systematic review*

Lin PY, Su KP. A meta-analytic review of double-blind, placebo-controlled trials of antidepressant efficacy of omega-3 fatty acids. *J Clin Psychiatry* 2007;68(7):1056-61.

*Reason: No efficacy data for placebo*

Lin SP, Long YM, Chen XH. The Effects of Statins on Infections after Stroke or Transient Ischemic Attack: A Meta-Analysis. *PloS one* 2015;10(7):e0130071.

*Reason: Patients did not have a mental disorder*

Lin SY, Su YX, Wu YC, et al. Management of paediatric obstructive sleep apnoea: A systematic review and network meta-analysis. *Int J Paediatr Dent* 2020;30(2):156-70.

*Reason: Patients did not have a mental disorder*

Litten RZ, Castle IJ, Falk D, et al. The placebo effect in clinical trials for alcohol dependence: an exploratory analysis of 51 naltrexone and acamprosate studies. *Alcohol Clin Exp Res* 2013;37(12):2128-37.

*Reason: No pooled effect size for placebo*

Locher C, Kossowsky J, Gaab J, et al. Moderation of antidepressant and placebo outcomes by baseline severity in late-life depression: A systematic review and meta-analysis. *J Affect Disord* 2015;181:50-60.

*Reason: Not the most information*

Ma D, Zhang Z, Zhang X, et al. Comparative efficacy, acceptability, and safety of medicinal, cognitive-behavioral therapy, and placebo treatments for acute major depressive disorder in children and adolescents: a multiple-treatments meta-analysis. *Curr Med Res Opin* 2014;30(6):971-95.

*Reason: No efficacy data for placebo*

Masi A, Lampit A, Glozier N, et al. Predictors of placebo response in pharmacological and dietary supplement treatment trials in pediatric autism spectrum disorder: a meta-analysis. *Transl Psychiatry* 2015;5(9):e640.

*Reason: Not the most information*

Matsusaki A, Kaneko M, Narukawa M. Meta-analysis of Placebo Response in Randomized Clinical Trials of Antipsychotic Drugs Using PANSS Focusing on Different Approaches to the Handling of Missing Data. 2018.

*Reason: Not the most information*

McCall WV, R. D'Agostino J, Dunn A. A meta-analysis of sleep changes associated with placebo in hypnotic clinical trials. *Sleep Med* 2003;4(1):57-62.

*Reason: Not a systematic review*

Meister R, Jansen A, Härter M, et al. Placebo and nocebo reactions in randomized trials of pharmacological treatments for persistent depressive disorder. A meta-regression analysis. *J Affect Disord* 2017;215:288-98.

*Reason: Not the most information*

Mora MS, Nestoriuc Y, Rief W. Lessons learned from placebo groups in antidepressant trials. *Philos Trans R Soc Lond B Biol Sci* 2011;366(1572):1879-88.

*Reason: Not a systematic review*

Nasir M, Li F, Courley S, et al. Meta-Analysis: Pediatric Placebo Response in Depression Trials Does Not Replicate in Anxiety and Obsessive-Compulsive Disorder Trials. 2021.

*Reason: Not a systematic review*

Naudet F, Millet B, Charlier P, et al. Which placebo to cure depression? A thought-provoking network meta-analysis. 2013.

*Reason: No efficacy data for placebo*

Nelson JC, Zhang Q, Deberdt W, et al. Predictors of remission with placebo using an integrated study database from patients with major depressive disorder. *Curr Med Res Opin* 2012;28(3):325-34.

*Reason: Not a systematic review*

Newcorn JH, Sutton VK, Zhang S, et al. Characteristics of placebo responders in pediatric clinical trials of attention-deficit/hyperactivity disorder. *J Am Acad Child Adolesc Psychiatry* 2009;48(12):1165-72.

*Reason: Not a systematic review*

Nierenberg AA, Østergaard SD, Iovieno N, et al. Predictors of placebo response in bipolar depression. *Int Clin Psychopharmacol* 2015;30(2):59-66.

*Reason: Not a systematic review*

Ogawa Y, Furukawa TA, Takeshima N, et al. Efficacy of antidepressants over placebo is similar in two-armed versus three-armed or more-armed randomized placebo-controlled trials. *International Clinical Psychopharmacology* 2018;33(2):66-72.

*Reason: No efficacy data for placebo*

Ona G, Bouso JC. Potential safety, benefits, and influence of the placebo effect in microdosing psychedelic drugs: A systematic review. *Neurosci Biobehav Rev* 2020;119:194-203.

*Reason: No efficacy data for placebo*

Oosterbaan DB, Balkom AJv, Spinhoven P, et al. The placebo response in social phobia. *J Psychopharmacol* 2001;15(3):199-203.

*Reason: Not a systematic review*

Papakostas GI, Fava M. Does the probability of receiving placebo influence clinical trial outcome? A meta-regression of double-blind, randomized clinical trials in MDD. 2009.

*Reason: Not a systematic review*

Parker G, Ricciardi T, Hadzi-Pavlovic D. Placebo response rates in trials of antidepressant drugs in adults with clinical depression: Increasing, decreasing, constant or all of the above? *Journal of Affective Disorders* 2020;271:139-44.

*Reason: Not a systematic review*

Posternak MA, Zimmerman M. Therapeutic effect of follow-up assessments on antidepressant and placebo response rates in antidepressant efficacy trials: meta-analysis. *Br J Psychiatry* 2007;190:287-92.

*Reason: Not a systematic review*

Rendell JM, Gijsman HJ, Bauer MS, et al. Risperidone alone or in combination for acute mania. *Cochrane Database Syst Rev* 2006;2006(1):CD004043.

*Reason: No efficacy data for placebo*

Rief W, Nestoriuc Y, Weiss S, et al. Meta-analysis of the placebo response in antidepressant trials. *J Affect Disord* 2009;118(1-3):1-8.

*Reason: Not a systematic review*

Rutherford BR, Mori S, Sneed JR, et al. Contribution of spontaneous improvement to placebo response in depression: a meta-analytic review. *J Psychiatr Res* 2012;46(6):697-702.

*Reason: Not a biological treatment*

Rutherford BR, Pott E, Tandler JM, et al. Placebo response in antipsychotic clinical trials: a meta-analysis. *JAMA Psychiatry* 2014;71(12):1409-21.

*Reason: Not the most information. No pooled effect size for placebo.*

Rutherford BR, Sneed JR, Tandler JM, et al. Deconstructing pediatric depression trials: an analysis of the effects of expectancy and therapeutic contact. *J Am Acad Child Adolesc Psychiatry* 2011;50(8):782-95.

*Reason: No efficacy data for placebo*

Salamone JD. A critique of recent studies on placebo effects of antidepressants: importance of research on active placebos. *Psychopharmacology (Berl)* 2000;152(1):1-6.

*Reason: Not a systematic review*

Schalkwijk S, Undurraga J, Tondo L, et al. Declining efficacy in controlled trials of antidepressants: effects of placebo dropout. *Int J Neuropsychopharmacol* 2014;17(8):1343-52.

*Reason: No efficacy data for placebo*

Scherrer B, Guiraud J, Addolorato G, et al. Baseline severity and the prediction of placebo response in clinical trials for alcohol dependence: A meta-regression analysis to develop an enrichment strategy. *Alcohol Clin Exp Res* 2021;45(9):1722-34.

*Reason: No efficacy data for placebo*

Sinyor M, Levitt AJ, Cheung AH, et al. Does inclusion of a placebo arm influence response to active antidepressant treatment in randomized controlled trials? Results from pooled and meta-analyses. 2010.

*Reason: No efficacy data for placebo*

Smet SD, Nikolin S, Moffa A, et al. Determinants of sham response in tDCS depression trials: a systematic review and meta-analysis. *Prog Neuropsychopharmacol Biol Psychiatry* 2021;109:110261.

*Reason: No efficacy data for placebo*

Stolk P, Berg MJT, Hemels ME, et al. Meta-analysis of placebo rates in major depressive disorder trials. *Ann Pharmacother* 2003;37(12):1891-9.

*Reason: No efficacy data for placebo*

Strawbridge R, Carter B, Marwood L, et al. Augmentation therapies for treatment-resistant depression: systematic review and meta-analysis. *Br J Psychiatry* 2019;214(1):42-51.

*Reason: Not the most information*

Sugarman MA, Kirsch I, Huppert JD. Obsessive-compulsive disorder has a reduced placebo (and antidepressant) response compared to other anxiety disorders: A meta-analysis. 2017.

*Reason: Not a systematic review*

Sysko R, Walsh BT. A systematic review of placebo response in studies of bipolar mania. *J Clin Psychiatry* 2007;68(8):1213-7.

*Reason: Not a systematic review*

Tang B, Barnes K, Geers A, et al. Choice and the Placebo Effect: A Meta-analysis. *Ann Behav Med* 2022;56(10):977-88.

*Reason: Not patients with a mental disorder*

Valerio MP, Szmulewicz AG, Martino DJ. A quantitative review on outcome-to-antidepressants in melancholic unipolar depression. *Psychiatry Res* 2018;265:100-10.

*Reason: No efficacy data for placebo*

Vambheim SM, Flaten MA. A systematic review of sex differences in the placebo and the nocebo effect. *J Pain Res* 2017;10:1831-39.

*Reason: No efficacy data for placebo*

Vöhringer PA, Ghaemi SN. Solving the antidepressant efficacy question: effect sizes in major depressive disorder. *Clin Ther* 2011;33(12):B49-61.

*Reason: Not a systematic review*

Walsh BT, Seidman SN, Sysko R, et al. Placebo response in studies of major depression: variable, substantial, and growing. *JAMA* 2002;287(14):1840-7.

*Reason: No efficacy data for placebo*

Weimer K, Colloca L, Enck P. Placebo effects in psychiatry: mediators and moderators. *Lancet Psychiatry* 2015;2(3):246-57.

*Reason: Not a systematic review*

Welge JA, P. E. Keck J. Moderators of placebo response to antipsychotic treatment in patients with schizophrenia: a meta-regression. *Psychopharmacology (Berl)* 2003;166(1):1-10.

*Reason: Not the most information*

Wernsdorff Mv, Loef M, Tuschen-Caffier B, et al. Effects of open-label placebos in clinical trials: a systematic review and meta-analysis. 2021.

*Reason: Patients did not have a mental disorder*

Yeung V, Sharpe L, Glozier N, et al. A systematic review and meta-analysis of placebo versus no treatment for insomnia symptoms. *Sleep Medicine Reviews* 2018;38:17-27.

*Reason: Patients did not have a mental disorder*

Yildiz A, Vieta E, Leucht S, et al. Efficacy of antimanic treatments: meta-analysis of randomized, controlled trials. *Neuropsychopharmacology* 2011;36(2):375-89.

*Reason: No efficacy data for placebo*

Yury CA, Fisher JE. Meta-analysis of the effectiveness of atypical antipsychotics for the treatment of behavioural problems in persons with dementia. *Psychother Psychosom* 2007;76(4):213-8.

*Reason: Not a systematic review*

Zhang N, Li Y, Lv Y, et al. Quantitative Comparison of the Efficacies of 5 First-Line Drugs for Primary Restless Leg Syndrome. *J Clin Pharmacol* 2019;59(9):1177-87.

*Reason: Not the most information*

## 2.2. Narrative Description of Meta-Analyses Reporting Placebo Effect Size

Here, we report the key findings by mental disorder in order of number of included RCT’s per meta-analysis per condition.

### 2.2.1. Major Depressive Disorder

We included four meta-analyses that reported a within-group placebo effect size in individuals with MDD. The largest of these meta-analyses was conducted in adults and included 347 clinical trials and 89,183 patients.^4^ The active treatment in included trials were concurrently approved and regulated antidepressant medications. The primary outcome of the included trials, reflected in the primary outcome of the meta-analysis, was reduction in depressive symptoms. The within-group placebo effect size was found to be g = 1·10 (95% CI [1·06, 1·15]). In contrast, the effect size for active medication was larger (g = 1·49, 95% CI [1·44, 1·53]). Results of sub-group analyses carried out by the authors showed that both placebo effect size and active medication effect size were significantly larger among studies that involved a single-blind placebo run-in period, but the between-group effect size was not significantly different (g = 0·33, 95% CI [0·29, 0·38] vs g = 0·34, 95% CI [0·30, 0·38]; p = 0·92).

In a separate meta-analysis of 17 RCTs comparing serotonergic medication (SSRI or SNRI) with placebo in children and adolescents with depression^5^ (*N* not reported), the within-group placebo effect size was large (g = 1·57, 95% CI [1·36, 1·78]). The active medication response was also large (g = 1·85, 95% CI [1·70, 2·00]). The primary outcome was the primary outcome of each study included in the meta-analysis, which involved a mixture of disorder-specific questionnaires or improvement on a general severity scale, such as the clinical global impression (CGI) scale.

We included one meta-analysis of patients with treatment-resistant depression (defined by the authors as non-response to ≥1 adequate antidepressant).^6^ This meta-analysis included 57 clinical trials in 5,606 patients. The active treatment in included studies was any medication used as in combination with standard treatment including antidepressants, antipsychotics, hormones, mood stabilisers, NMDA antagonists, stimulants, and vitamins. The primary outcome was improvement in depressive symptoms on a validated scale, with preference for clinician-rated symptoms. Within-group placebo effect size was g = 0·89 (95% CI [0·81, 0·98]). There was no pooled analysis of active treatment effect size, but individual treatment modalities were explored and the confidence intervals for only six of these did not cross those of placebo.

We also included a meta-analysis of 61 neuromodulation trials (repetitive transcranial magnetic stimulation or transcranial direct current stimulation) in 1,328 patients with MDD.^7^ The primary outcome was change in depressive symptoms, but it was not specified whether preference was given to clinician or patient-rated symptoms. The effect size of sham neuromodulation was g = 0·80 (95% CI [0·65, 0·95]). However, some of these clinical trials included patients with treatment-resistant depression. Sub-group analyses showed that the placebo effect size reduced as number of previously failed treatment trials increased. The effect size was g = 1·28 (95% CI [0·47, 2·97]) for non-treatment-resistant patients, g = 0·67 (95% CI [0·06, 1·28]) in patients non-responsive after one or more treatment trials, and g = 0·50 (95% CI [0·03, 0·99]) in patients non-responsive after two or more treatment trials. This meta-analysis did not include an analysis of active treatment effect size.

### 2.2.2. Anxiety Disorders

We included four meta-analyses reporting placebo effect sizes in anxiety disorders: one in panic disorder^8^, one reporting an effect size in patients with generalised anxiety disorder (GAD) and social anxiety disorder (SAD)^9^, one across all anxiety disorders in children and adolescents^5^, and one in older adults.^10^

First, we included a meta-analysis of 43 trials encompassing 2,392 patients with panic disorder. Active treatments in included trials consisted of benzodiazepines, antidepressants, and gabapentinoids. Overall placebo effect size was medium (d = 0·57, 95% CI [0·50, 0·64]).^8^ Secondary analyses conducted by the authors revealed that placebo effect size varied depending on the outcome measure, for example, larger effects were seen with clinician-rated outcomes (d = 0·75, 95% CI [0·66, 0·84]) compared with self-rated outcomes (d = 0·35, 95% CI [0·28, 0·43]) or objective measures such as blood pressure (d = 0·19, 95% CI [-0·06, 0·45]). Placebo effect size was also significantly larger on intention-to-treat (d = 0·64, 95% CI [0·55, 0·74]) compared with per-protocol analyses (d = 0·49, 95% CI [0·41, 0·58]). Pooled active treatment effect size was not reported.

We additionally included a meta-analysis reporting placebo effect sizes in patients with GAD (k = 39) or with SAD (k = 37) based on pre-post change in Hamilton Rating Scale for Anxiety or Liebowitz Social Anxiety Scale.^9^ Active treatments were those known to be effective for the treatment of anxiety disorders: the SSRIs citalopram, escitalopram, fluoxetine, fluvoxamine, paroxetine and sertraline, the SNRIs duloxetine and venlafaxine, pregabalin, the TCAs imipramine, clomipramine, the benzodiazepines alprazolam, bromazepam, clobazam, clonazepam, delorazepam, diazepam and lorazepam, the antihistamine hydroxyzine, the irreversible monoamine oxidase inhibitor phenelzine, the reversible inhibitor of monoamine oxidase A moclobemide, and the antipsychotic quetiapine. Within-group placebo effect size was large in GAD (d = 1·85, 95% CI [1·61, 2·09]) and in SAD (d = 0·94, 95% CI [0·77, 1·12]). This meta-analysis did not include results regarding active treatment effect size.

In a meta-analysis of trials comparing SSRIs or SNRIs with placebo conducted in children and adolescents, results were pooled across all anxiety disorders, and placebo effect size was found to be large (g = 1·03, 95% CI [0·84, 1·21]).^5^ Medication effect size was large (g = 1·68, 95% CI [1·56, 1·79]) with a significant between-group effect size of g = 0·56 (95% CI [0·40, 0·72]).

We included one meta-analysis of randomised-controlled trials in older adults (mean or median age ≥60 years) with GAD or panic disorder (k = 13, N = 1,273). The active treatments in included trials were abecarnil, alpidem, alprazolam, buspirone, carbamazepine, fluvoxamine, imipramine, nefazodone, nortriptyline, oxazepam, sertraline, and venlafaxine. The placebo effect size was large (d = 1·06, 95% CI [0·71, 1·42]).^10^ Secondary analyses suggested this effect size was similar when considering both self-rated (d = 1·09, 95% CI [0·79, 1·38]) or clinician-rated (d = 1·13, 95% CI [0·74, 1·53]) outcomes. In addition, active medication effect size was large (d = 1·76, 95% CI [1·58, 1·95]) and significantly superior to placebo (between-group effect size d = 0·83, 95% CI [0·52, 1·14]).

### 2.2.3. Attention-Deficit/Hyperactivity Disorder

We one meta-analysis reporting placebo effect size in patients with ADHD.^11^ This meta-analysis predominantly included randomised-controlled trials in children and adolescents, but also some trials with adult patients. A meta-regression analysis showed that there was no significant effect of age on placebo effect size. Active medications included amphetamines (including lisdexamfetamine), atomoxetine, bupropion, clonidine, guanfacine, methylphenidate (including dexmethylphenidate), and modafinil. The primary outcome was clinician-rated improvement in ADHD symptoms. The placebo effect size for this primary outcome was medium to large (SMC = 0·75, 95% CI [0·67, 0·83]). Secondary outcomes included teacher, parent, and self-ratings of improvement in ADHD symptoms. The placebo effect size was smaller for these secondary outcomes, with teacher ratings having the smallest effect size (SMC = 0·36), parent ratings next the largest (SMC = 0·43), and self-ratings having the largest effect size (SMC = 0·66). All effect sizes were statistically significant. Effect sizes for active medication were not reported in this meta-analysis. However, correlations between placebo effect size and drug-placebo difference were explored. There was a significant negative relationship between placebo effect size and drug-placebo difference (-0·56, p < 0·01) for self-rated outcomes, but all other correlations were not significant.

### 2.2.4. Schizophrenia Spectrum Disorders

We included two meta-analyses reporting within-group placebo effect sizes in schizophrenia spectrum disorders.^12,13^ The largest of these included 61 trials and 14,787 patients.^12^ Active treatments included both approved and experimental antipsychotic medications. The primary outcome was reduction in psychotic symptoms assessed via the Brief Psychiatric Rating Scale (BPRS) or Positive and Negative Syndrome Scale (PANSS). The pooled placebo effect size was small to medium (standardised mean change = 0·33, 95% CI [0·22, 0·44]). There was no pooled estimate of active effect size. The second meta-analysis explored placebo response in patients with negative symptoms of schizophrenia.^13^ Twenty-five trials including 4,391 patients were meta-analysed with improvement in negative symptoms as the primary outcome. Both add-on trials and monotherapy trials were included, but the exact medications included were not reported. The placebo effect size was medium (d = 0·64, 95% CI [0·46, 0·83]). There was no pooled estimate of active effect size.

### 2.2.5. Restless Legs Syndrome

We included one meta-analysis involving patients with RLS.^14^ This meta-analysis encompassed 85 randomised-controlled trials and 5,046 patients. The most studied medications were ropinirole, pramipexole, rotigotine, and gabapentin-enacarbil. The primary outcome was reduction in symptoms measured through any rating scale assessing severity or disability. The placebo effect size for this outcome was large (g = 1·41, 95% CI [1·25, 1·56]). Secondary outcomes included objective measures, such as periodic limb movements index, and subjective measures, such as quality of life. Placebo effect sizes for these outcomes were smaller, ranging from g = 0·02 to g = 0·67. In particular, objective outcomes demonstrated low heterogeneity (I^2^ = 0%) and small placebo effect sizes (g = 0·02 to g = 0·24). Effect sizes for active medication were not reported in this meta-analysis.

### 2.2.6. Autism Spectrum Disorders

One meta-analysis met inclusion criteria exploring placebo effect size in patients with autism spectrum disorders.^2^ This meta-analysis encompassed 86 clinical trials with 5,365 patients. The majority (87%) of patients were children and adolescents. Trials comparing any medication or dietary supplements with placebo were included. Medications included amantadine, arbaclofen, aripiprazole, atomoxetine, balovaptan, bumetanide, buspirone, citalopram, divalproex, donepezil, fenfluramine, fluoxetine, fluvoxamine, guanfacine, haloperidol, oral human immunoglobulin, lamotrigine, levetiracetam, lurasidone, mecamylamine, memantine, mirtazapine, naltrexone, olanzapine, oxytocin, riluzole, risperidone, sertraline, simvastatin, tianeptine, and valproate. Primary outcomes were reductions in social-communication difficulties, repetitive-restrictive behaviours, and overall measures of core symptoms, measured through standard questionnaires. Placebo effect sizes for these primary outcomes were small (ranging from SMC = 0·23 to SMC = 0·36). In secondary analyses, the authors explored whether there was an effect of rater (teacher, clinician, caregiver) on placebo effect size. This rater stratification did not substantially alter placebo effect sizes. Likewise, there was no relationship between age and placebo effect size. Effect sizes for active medication were not reported.

### 2.2.7. Obsessive-Compulsive Disorder

We included one meta-analysis exploring placebo effect size in OCD.^15^ This encompassed 49 clinical trials in 1,993 patients. Active interventions involved biological treatments including medications (atorvastatin, citalopram, clomipramine, clonazepam, echium amoenum, escitalopram, fluoxetine, fluvoxamine, glycine powder, imipramine, inositol, nortriptyline, N-acetylcysteine, oxytocin, paroxetine, phenelzine, sertraline, St John’s Wort, trazodone), repetitive transcranial magnetic stimulation, and gamma ventral capsulotomy. The primary outcome was change in ‘OCD-specific’ outcomes (e.g. Y-BOCS), which exhibited a small placebo effect size (d = 0·32, 95% CI [0·22, 0·41]). Subgroup analyses performed by the authors showed that placebo effect size significantly differed between children & adolescents (d = 0·45, 95% CI [0·35, 0·56]) and adults (d = 0·27, 95% CI [0·15, 0·38]); and between intention-to-treat (d = 0·43, 95% CI [0·28, 0·58]) and per-protocol analyses (d = 0·20, 95% CI [0·11, 0·29]). Pooled effect sizes for active medication were not calculated.

### 2.2.8. Alcohol Use Disorder

One meta-analysis met inclusion criteria exploring placebo effect size in AUD.^16^ This meta-analysis included 47 clinical trials, but sample size and active treatments were not reported. The primary outcome was not clearly defined, but the authors reported that the placebo effect size was large (g = 0·90, 95% CI [0·70, 1·09]). Effect sizes for active medication were not calculated.

### 2.2.9. Primary Insomnia

We included one meta-analysis reporting placebo effect size in primary chronic insomnia.^17^ In this meta-analysis, placebo and active effect sizes of medication were compared in a number of objective (recorded through polysomnography) and subjective (assessed through sleep diaries or questionnaires) outcomes. The meta-analysis encompassed 32 clinical trials and 3,969 patients. The active treatment arms were described in only broad terms: 17 hypnotic drugs, 6 antidepressants, 8 antiepileptics, 7 benzodiazepines, 1 antihistamine, 2 GABA receptor modulators, 1 hormone, 1 melatonin receptor agonist, 1 narcotic, 1 neuropeptide, 1 progesterone receptor antagonist, and 2 valerian preparations. The placebo effect sizes for the primary outcomes, objective and subjective sleep onset latency, were significant and small (g = 0·35, 95% CI [0·28, 0·35] and g = 0·29, 95% CI [0·19, 0·39], respectively). The effect sizes for active medication in these outcomes were medium (g = 0·55, 95% CI [0·46, 0·63] and g = 0·45, 95% CI [0·34, 0·57], respectively). The authors calculated the proportion of active medication response explained by placebo response, which was 64% for both these primary outcomes. In all secondary outcome measures, placebo effect sizes were significant and small (g ranged from 0·25 to 0·43), with no significant difference in effect size between objective and subjective outcomes. The effect sizes for active medication were mostly medium (g ranged from 0·29 to 0·79).

### 2.2.10. Intellectual Disability

We included one meta-analysis of patients with genetically-determined intellectual disability (Fragile X, Down’s, Prader-Willi, and Williams syndromes).^18^ This meta-analysis consisted of 22 clinical trials and 721 patients. The medications included in the active treatment arms were not described. The primary outcome was overall reduction in symptoms, calculated by combining multiple effect sizes from individual studies for outcomes evaluating behavioural or cognitive-developmental functions. This analysis showed a small to medium placebo effect size of g = 0·47 (95% CI [0·18, 0·76]). However, once outlier studies were removed, placebo effect size reduced to g = 0·20. Effect sizes for active medication were numerically larger (primary outcome g = 0·68, 95% CI [0·34, 1·01]) but not significantly different. The authors conducted a number of secondary and subgroup analyses. First, placebo effect size was numerically higher for subjective compared with objective outcomes (g = 0·56 vs g = 0·43), although this difference was not statistically significant (Q_(1)_ = 0·16, p = 0·69). Second, there was a significant difference in placebo effect size (Q_(6)_ = 13·29, p = 0·04) and medication effect size (Q_(6)_ = 17·35, p = 0·01) depending on the mental process the outcome was measuring. Placebo response was significant for cognitive and developmental (k = 17, g = 0·31), abnormal behaviour (k = 7, g = 0·28), autistic traits (k = 3, g = 0·34), and CGI (k = 3, g = 2·22) outcomes. Placebo response was not significant for measures related to attention, language, or memory. There were no significant differences between placebo and medication response on any measure. Third, placebo responses were significantly larger in patients with higher IQ (IQ ranged from 20 to 65; Q_(2)_ = 7·48, p = 0·02). This effect was not present for active medication response. Fourth, both placebo and drug response was larger in younger participants (Q’s >5·2, p’s <0·02). Fifth, placebo effect size was negligible in patients with comorbid dementia (g = -0·05), but medium in patients without dementia (g = 0·51). Finally, there was no effect of year of publication on placebo (Q_(1)_ = 0·06, p = 0·81) or medication effect size (Q_(1)_ = 1·34, p = 0·25).

## 2.3. AMSTAR-2 Ratings

# 3. eDiscussion

We found several correlates of placebo effect size or response rate reported in meta-analyses. Publication year was frequently explored across clinical trials. Interestingly, later publication year was associated with greater placebo response in many mental disorders. However, in MDD, placebo effect size appeared to stabilise since approximately 1991 onwards.^19^ The authors of this meta-analysis theorised that this was perhaps the result of several methodological variables becoming standard, e.g. fixed dosing, standard trial durations, etc.^19^ Similar analyses assessing whether there is a ‘break point’ in the association with publication year have not been conducted in other mental disorders. Intriguingly, publication year was *negatively* associated with placebo response in ADHD.^11^ The reasons for this opposite finding are unknown, but more recently more trials have been conducted in adult patients with ADHD, and younger age appears correlated with increased placebo responses as demonstrated in schizophrenia^12,20^ and OCD.^15^ In addition, increased sample size and a higher number of study sites were associated with placebo effect size across several mental disorders, including MDD, panic disorder, schizophrenia spectrum disorders, autism spectrum disorders, and OCD.^2,8,15,19–21^ One possible explanation for this finding is that a larger sample increases the probability of including patients who exhibit greater volatility in symptoms and therefore the possibility of larger placebo effect sizes.^22^ Another possibility is that with more sites, there is increased probability of including a site with reduced expertise or training in trial procedures and outcome measures, leading to increased ‘noise’ (variance) in the data. These possibilities have yet to be explicitly investigated.

Increased baseline illness severity was associated with increased placebo responses in schizophrenia spectrum disorders, ADHD, and AUD.^11,12,16^ This could represent a statistical artefact, driven either by the artefactual correlation between baseline illness severity and magnitude of symptom improvement^23^, or regression to the mean, or baseline symptom inflation. However, in other studies, *lower* baseline illness severity has been associated with increased placebo response in psychosis, SAD, depression, and ADHD.^24–27^ The reasons for these discrepancies are not clear. Potentially, baseline illness severity represents another unmeasured latent variable in certain studies, such as illness duration or different baseline expectations^25^ (see below). To inform the conduct of future RCTs in mental disorders, further research to explore the relationship between baseline illness severity and response to both medication and placebo is warranted.

We found that increased trial duration conferred a variable impact on placebo effect size across disorders. Increased trial duration was associated with *larger* placebo effect size in major depression^19^, while in schizophrenia spectrum conditions, it was associated with *smaller* placebo effect size.^12,13^ A potential explanation for this variable impact could be a statistical artefact related to the natural history of a disorder, i.e., disorders in which there is greater natural fluctuation in severity, such as depression^28^, will show larger placebo effect sizes with longer trials compared with disorders with lower natural severity fluctuation (e.g., schizophrenia^29^). Intuitively, cohorts with a shorter duration of illness (for example, younger populations) are less likely to have persistent symptoms and so are more likely to show improvements. Indeed, where data were available, the placebo effect size was larger in children and adolescents than in adults in MDD and in OCD.^5,15^ On the other hand, the placebo effect size for negative symptoms seemed to be *larger* than in positive symptoms in schizophrenia spectrum conditions in the meta-analyses we included in this review.^13^ This may possibly be due to responsiveness of negative symptoms to familiarity with, and non-specific interventions by, study staff as part of the attention provided during a clinical trial. In addition, another aspect that might be important, at least in the case of major depressive disorder, is the effect of repeated measurement of symptoms. There is evidence to suggest that rating scales for depression do not measure the same underlying construct(s) in the same way over time, with scales likely becoming more reliable over time.^30,31^ This effect might be more pronounced with longer clinical trials, confounding the interpretation of symptom reduction in placebo, and potentially medication, groups. Indeed, we found that where objective measures were taken (in OCD, primary insomnia, and RLS), these were associated with smaller placebo effect sizes than with subjective outcomes.^14,15,17^ Further work is needed to understand the relationship between trial duration and placebo effect size in mental disorders.

It is noteworthy that low risk of bias in allocation concealment was associated with increased placebo effect size in autism spectrum disorders.^2^ Success in blinding both patients and raters is thought to potentially influence placebo response in MDD, possibly due to unblinding leading to reduced expectations in the placebo arm of a trial.^32,33^ Similar effects are also being actively explored in trials of psychedelic compounds.^34,35^ However, blinding success is little reported.^33,36^ How presence or lack of bias influences the placebo effect within clinical trials is an important issue that needs further exploration.

Given that the trial-level correlates in these meta-analyses were identified *post-hoc* (i.e., after the clinical trials included in the meta-analysis were conducted), it is possible that relationships between these correlates and placebo response result from interactions with unidentified latent variables. For example, larger numbers of active treatment arms was a significant predictor of placebo response in clinical trials for negative symptoms of schizophrenia.^13^ This variable has also been associated with placebo response in trials for MDD.^37^ Potentially, the higher likelihood of being randomised to active treatment results in increased expectations of therapeutic benefit, leading to larger placebo effect size.^38^ This association with greater expectation effects might also explain why flexible dosing is associated with increased placebo effect size in MDD (higher dose might mean higher expectations).^19^ Meta-analysis of individual patient data might be more effective for identifying such latent variables that cannot be assessed using summary statistics such as the mean.^23,39^

# 4. eReferences

1 Krnic Martinic M, Pieper D, Glatt A, Puljak L. Definition of a systematic review used in overviews of systematic reviews, meta-epidemiological studies and textbooks. *BMC Med Res Methodol* 2019; **19**: 203.

2 S. Siafis, O. Ç?ray, J. Schneider-Thoma, *et al.* Placebo response in pharmacological and dietary supplement trials of autism spectrum disorder (ASD): systematic review and meta-regression analysis. *Mol Autism* 2020; **11**: 66.

3 Masi A, Lampit A, Glozier N, Hickie IB, Guastella AJ. Predictors of placebo response in pharmacological and dietary supplement treatment trials in pediatric autism spectrum disorder: a meta-analysis. *Transl Psychiatry* 2015; **5**: e640–e640.

4 Scott AJ, Sharpe L, Quinn V, Colagiuri B. Association of Single-blind Placebo Run-in Periods With the Placebo Response in Randomized Clinical Trials of Antidepressants: A Systematic Review and Meta-analysis. *JAMA Psychiatry* 2022; **79**: 42.

5 C. Locher, H. Koechlin, S. R. Zion, *et al.* Efficacy and Safety of Selective Serotonin Reuptake Inhibitors, Serotonin-Norepinephrine Reuptake Inhibitors, and Placebo for Common Psychiatric Disorders Among Children and Adolescents: A Systematic Review and Meta-analysis. *Jama Psychiatry* 2017; **74**: 1011–20.

6 F. Scott, E. Hampsey, S. Gnanapragasam, *et al.* Systematic review and meta-analysis of augmentation and combination treatments for early-stage treatment-resistant depression. *J Psychopharmacol* 2022; : 2698811221104058.

7 L. B. Razza, A. H. Moffa, M. L. Moreno, *et al.* A systematic review and meta-analysis on placebo response to repetitive transcranial magnetic stimulation for depression trials. *Prog Neuropsychopharmacol Biol Psychiatry* 2018; **81**: 105–13.

8 M. Ahmadzad-Asl, F. Davoudi, S. Mohamadi, *et al.* Systematic review and meta-analysis of the placebo effect in panic disorder: Implications for research and clinical practice. *Aust N Z J Psychiatry* 2022; **56**: 1130–41.

9 Bandelow B, Reitt M, Röver C, Michaelis S, Görlich Y, Wedekind D. Efficacy of treatments for anxiety disorders: a meta-analysis. *Int Clin Psychopharmacol* 2015; **30**: 183–92.

10 M. Pinquart, P. R. Duberstein. Treatment of anxiety disorders in older adults: a meta-analytic comparison of behavioral and pharmacological interventions. *Am J Geriatr Psychiatry* 2007; **15**: 639–51.

11 Faraone SV, Newcorn JH, Cipriani A, *et al.* Placebo and nocebo responses in randomised, controlled trials of medications for ADHD: a systematic review and meta-analysis. *Mol Psychiatry* 2022; **27**: 212–9.

12 Agid O, Siu CO, Potkin SG, *et al.* Meta-Regression Analysis of Placebo Response in Antipsychotic Trials, 1970–2010. *Am J Psychiatry* 2013; **170**: 1335–44.

13 P. Czobor, B. Kakuszi, I. Bitter. Placebo Response in Trials of Negative Symptoms in Schizophrenia: A Critical Reassessment of the Evidence. *Schizophr Bull* 2022; **48**: 1228–40.

14 M. A. Silva, G. S. Duarte, R. Camara, *et al.* Placebo and nocebo responses in restless legs syndrome: A systematic review and meta-analysis. *Neurology* 2017; **88**: 2216–24.

15 S. Mohamadi, M. Ahmadzad-Asl, S. A. Nejadghaderi, *et al.* Systematic Review and Meta-Analysis of the Placebo Effect and its Correlates in Obsessive Compulsive Disorder. *Can J Psychiatry* 2022; : 7067437221115029.

16 Del Re AC, Maisel N, Blodgett J, Wilbourne P, Finney J. Placebo group improvement in trials of pharmacotherapies for alcohol use disorders: a multivariate meta-analysis examining change over time. *J Clin Psychopharmacol* 2013; **33**: 649.

17 A. Winkler, W. Rief. Effect of Placebo Conditions on Polysomnographic Parameters in Primary Insomnia: A Meta-Analysis. *Sleep* 2015; **38**: 925–31.

18 A. Curie, K. Yang, I. Kirsch, *et al.* Placebo Responses in Genetically Determined Intellectual Disability: A Meta-Analysis. *PloS One* 2015; **10**: e0133316.

19 Furukawa TA, Cipriani A, Atkinson LZ, *et al.* Placebo response rates in antidepressant trials: a systematic review of published and unpublished double-blind randomised controlled studies. *Lancet Psychiatry* 2016; **3**: 1059–66.

20 S. Leucht, A. Chaimani, C. Leucht, *et al.* 60 years of placebo-controlled antipsychotic drug trials in acute schizophrenia: Meta-regression of predictors of placebo response. *Schizophr Res* 2018; **201**: 315–23.

21 R. Meister, M. Abbas, J. Antel, *et al.* Placebo response rates and potential modifiers in double-blind randomized controlled trials of second and newer generation antidepressants for major depressive disorder in children and adolescents: a systematic review and meta-regression analysis. *Eur Child Adolesc Psychiatry* 2020; **29**: 253–73.

22 Leucht S, Leucht C, Huhn M, *et al.* Sixty Years of Placebo-Controlled Antipsychotic Drug Trials in Acute Schizophrenia: Systematic Review, Bayesian Meta-Analysis, and Meta-Regression of Efficacy Predictors. *Am J Psychiatry* 2017; **174**: 927–42.

23 Stone MB, Yaseen ZS, Miller BJ, Richardville K, Kalaria SN, Kirsch I. Response to acute monotherapy for major depressive disorder in randomized, placebo controlled trials submitted to the US Food and Drug Administration: individual participant data analysis. *BMJ* 2022; **378**: e067606.

24 Stein DJ, Baldwin DS, Dolberg OT, Despiegel N, Bandelow B. Which factors predict placebo response in anxiety disorders and major depression? An analysis of placebo-controlled studies of escitalopram. *J Clin Psychiatry* 2006; **67**: 1741–6.

25 Weimer K, Colloca L, Enck P. Placebo eff ects in psychiatry: mediators and moderators. *Lancet Psychiatry* 2015; **2**: 246–57.

26 Rutherford BR, Pott E, Tandler JM, Wall MM, Roose SP, Lieberman JA. Placebo Response in Antipsychotic Clinical Trials: A Meta-analysis. *JAMA Psychiatry* 2014; **71**: 1409.

27 Waxmonsky JG, Waschbusch DA, Glatt SJ, Faraone SV. Prediction of Placebo Response in 2 Clinical Trials of Lisdexamfetamine Dimesylate for the Treatment of ADHD. *J Clin Psychiatry* 2011; **72**: 15027.

28 Cuijpers P, Stringaris A, Wolpert M. Treatment outcomes for depression: challenges and opportunities. *Lancet Psychiatry* 2020; **7**: 925–7.

29 Bromet EJ, Fennig S. Epidemiology and natural history of schizophrenia. *Biol Psychiatry* 1999; **46**: 871–81.

30 Fokkema M, Smits N, Kelderman H, Cuijpers P. Response shifts in mental health interventions: An illustration of longitudinal measurement invariance. *Psychol Assess* 2013; **25**: 520–31.

31 Fried EI, Van Borkulo CD, Epskamp S, Schoevers RA, Tuerlinckx F, Borsboom D. Measuring depression over time . . . Or not? Lack of unidimensionality and longitudinal measurement invariance in four common rating scales of depression. *Psychol Assess* 2016; **28**: 1354–67.

32 Goodwin GM, Croal M, Marwood L, Malievskaia E. Unblinding and demand characteristics in the treatment of depression. *J Affect Disord* 2023; **328**: 1–5.

33 Scott AJ, Sharpe L, Colagiuri B. A systematic review and meta-analysis of the success of blinding in antidepressant RCTs. *Psychiatry Res* 2022; **307**: 114297.

34 Szigeti B, Nutt D, Carhart-Harris R, Erritzoe D. The difference between ‘placebo group’ and ‘placebo control’: a case study in psychedelic microdosing. *Sci Rep* 2023; **13**: 12107.

35 Lii TR, Smith AE, Flohr JR, *et al.* Randomized trial of ketamine masked by surgical anesthesia in patients with depression. *Nat Ment Health* 2023; : 1–11.

36 Lin Y-H, Sahker E, Shinohara K, *et al.* Assessment of blinding in randomized controlled trials of antidepressants for depressive disorders 2000–2020: A systematic review and meta-analysis. *eClinicalMedicine* 2022; **50**: 101505.

37 G. I. Papakostas, M. Fava. Does the probability of receiving placebo influence clinical trial outcome? A meta-regression of double-blind, randomized clinical trials in MDD. 2009.

38 Huneke NTM, van der Wee N, Garner M, Baldwin DS. Why we need more research into the placebo response in psychiatry. Psychol Med. 2020; **50**: 2317–23.

39 Welten CCM, Koeter MWJ, Wohlfarth T, *et al.* Placebo response in antipsychotic trials of patients with acute mania. *Eur Neuropsychopharmacol* 2015; **25**: 1018–26.
